# Crosstalk between NRP1 and autophagy in the tumor microenvironment: from molecular mechanisms to therapeutic targeting

**DOI:** 10.3389/fimmu.2026.1786502

**Published:** 2026-03-23

**Authors:** You Wang, Hong Ma, Linru Yang, Xiangfei Wang, Guorong Qi, Jichun Ma, Mingxu Da, Yaoqi Li

**Affiliations:** 1 The First School of Clinical Medicine, Gansu University of Chinese Medicine, Lanzhou, China; 2 Department of Interventional Oncology, Gansu Provincial Hospital, Lanzhou, China; 3 Department of Surgical Oncology, Gansu Provincial Hospital, Lanzhou, China

**Keywords:** autophagy, drug resistance, immunotherapy, NRP1, targeted therapy, tumor microenvironment

## Abstract

Neuropilin-1 (NRP1) is overexpressed in various malignant solid tumors, modulating the tumor microenvironment (TME) via multiple mechanisms to promote immune suppression, angiogenesis, and epithelial-mesenchymal transition (EMT), ultimately resulting in poor patient survival. Autophagy, a highly conserved cellular self-degradation process, plays a stage-dependent, bidirectional role in cancer. At early stages it suppresses tumorigenesis by clearing damaged cellular components, whereas at advanced stages it supports tumor survival under stress and thereby enhances proliferation, invasiveness, and therapy resistance. The interplay between NRP1 and autophagy in the TME is characterized by reciprocal regulation: NRP1 activates certain pathways to regulate autophagy, whereas autophagy induction promotes NRP1 degradation. This bidirectional interplay directly governs tumor progression and therapy resistance. Although prior studies have provided some clues about their interaction, the regulatory network and the precise mechanisms linking NRP1 and autophagy in the TME remain incompletely characterized. Precision therapies targeting the NRP1-autophagy axis still face multiple obstacles. This review synthesizes data from the atlas cancer genome (TCGA), the genotype-tissue expression (GTEx) database, and the human autophagy database (HADb) to explore associations between NRP1 and autophagy-related gene (ATG) in expression and prognosis, elucidate NRP1-autophagy interaction mechanisms and therapeutic opportunities and challenges in targeting the NRP1-autophagy axis. Pan-cancer analysis showed significant upregulation of NRP1 in 10 solid tumor types and revealed co-expression relationships between NRP1 and 340 ATGs. Among these, co-expression patterns involving genes such as CXCR4 and HSPA5 had significant prognostic value in gastric cancer and glioblastoma. This review systematically explores the panoramic regulatory framework of the NRP1-autophagy axis in the tumor immune microenvironment through bidirectional regulation of activating immunity and inhibitory immunity at the pan-cancer level. It fills the gap in the systematic summary of the NRP1-autophagy axis in regulating the dynamics of the tumor immune microenvironment, and provides a theoretical basis for the clinical translation of combination chemotherapy and immunotherapy targeting the NRP1-autophagy axis.

## 1 Introduction

Over the last decade, significant progress has been made in the diagnosis and treatment of malignant tumors, with the introduction of surgical, chemotherapy, radiotherapy, targeted therapy, and immunotherapy revolutionizing clinical practice. However, tumor drug resistance, recurrence, and metastasis remain major barriers to improving patient survival. Malignant tumors are not merely isolated clusters of proliferating cancer cells but form a complex, dynamic “organ”—TME—consisting of neoplastic cells and non-neoplastic components, including immune cells (regulatory T cells, M2 macrophages, cytotoxic CD8^+^ T cells), cancer-associated fibroblasts (CAFs), endothelial cells (ECs), extracellular matrix (ECM), and cytokines (1). Hypoxia, nutrient deficiency, and inflammatory factors in the TME affect tumor initiation and progression by regulating signal pathway transduction (2). Infiltrating immune cells mediate immune suppression to form a suppressive tumor immunological microenvironment (STIME). CAFs contribute to tumor drug resistance by mediating physical barriers, immune rejection, metabolic competition, and abnormal vascular structure formation (3). Although the TME synergistically drives multidrug resistance through multiple mechanisms, its inherent plasticity paradoxically offers promising prospects for targeted interventions to reverse drug resistance (3, 4).

In 1997, NRP1 was first discovered to participate in neuronal guidance and angiogenesis as a receptor for semaphorin-3A (SEMA3A) during nervous system development (5, 6). Subsequent studies have found that NRP1, as a “co-receptor” of transmembrane glycoproteins, is abnormally expressed in various tumors. In the TME, NRP1 regulates tumor proliferation, migration, and invasion by modulating immune function and its overexpression is closely associated with patients’ prognosis (7). NRP1 binds to the extracellular matrix protein such as fibronectin-1 (FN-1) to promote tumor metastasis by EMT and maintain characteristics of cancer stem cells (CSCs) (8), and it activates multiple signaling pathways including EGFR/AKT, FAK/PI3K/AKT, TGF-β/SMAD and HIF-1α (9–12). NRP1 overexpression is also associated with increased infiltration of Tregs and M2-like macrophages, inducing STIME formation to accelerate tumor growth and immune escape (13–15). Tumor-associated macrophage (TAM) polarization, a key regulator of tumor immune escape, is also modulated by NRP1 signaling (16).

Autophagy plays a “double-edged sword” role in tumors. In the early stage, it inhibits tumorigenesis by degrading damaged components, while in the late stage, it is exploited by tumor cells to promote tumor proliferation and metastasis (17–26). Autophagy in tumor cells can inhibit antigen presentation and participate in immune regulation (17, 18, 27); it is also correlated with the drug resistance mechanism in cancer therapy (17, 19, 21). Altering autophagic activity can regulate the efficacy of chemotherapy, targeted therapy, and immunotherapy (28–30). Autophagy is also involved in TME remodeling, metabolic regulation, inflammatory responses, genome repair, cell cycle control, and CSCs homeostasis maintenance, all of which contribute to tumor recurrence and metastasis (17, 19, 22, 23).

Recent studies indicate that NRP1 negatively regulates autophagic activity (31),while autophagy, in turn, regulates NRP1 expression and associated drug resistance (32). However, the mechanism of interaction between NRP1 and autophagy in the TME remains undefined. An in-depth exploration of the complex interaction mechanism between NRP1 and autophagy in the TME contributes to uncovering novel mechanisms of tumorigenesis, development, and drug resistance, and provides theoretical support for the clinical translation of precision therapies targeting the NRP1-autophagy axis.

## 2 Biological functions of NRP1 in tumors

### 2.1 Structural and molecular features of NRP1

**Figure 1:**
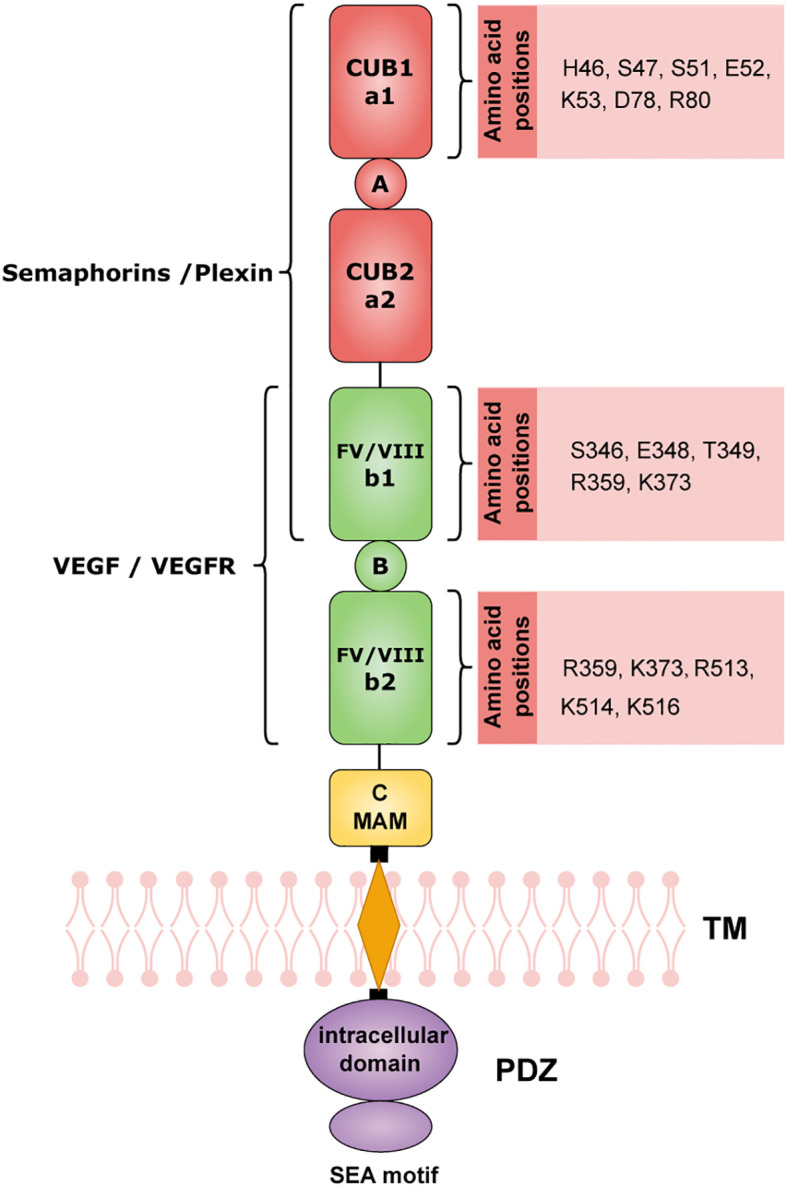
The core structure of NRP1. NRP1 has a long extracellular domain, a transmembrane domain, and a short intracellular domain. The extracellular domain includes N-terminal domain A (CUB) a1/a2, homologous domain B (b1/b2) from coagulation factors V and VIII, and C-terminal MAM domain. The TM domain contains about 25 amino acids. The intracellular domain contains a PDZ homology domain-binding domain (SEA motif). The structural domains are based on UniProt (ID: O14786, accessed on Oct 20, 2025) and published literature (34, 35). Created with BioGDP.com (158).

NRP1 is a single-transmembrane glycoprotein containing approximately 860 amino acids (molecular weight: 130–140 kDa), which plays a crucial role as a co-receptor in various physiological and pathological processes, especially in the TME. The structure of NRP1 consists of three major modules: the extracellular domain, transmembrane domain, and intracellular domain (**Figure 1**). The unique structure of NRP1 enables it to receive multiple signals (**Table 1**).

**Table 1:** NRP1 domains and their correlated ligands in cancer.

Domain type	Subdomains	Key amino acid positions	Ligand	Refs
Extracellular domain	A(CUB, a1/a2)	a1 :H46, S47, S51, E52, K53, D79, R80	SEMA family :SEMA3(A, B, C, D, F), SEMA4A;Plexin-A (1, 2, 3, 4); Plexin D1	(34, 35, 39, 40, 51)
	B(FV/VIII, b1/b2)	b1 :S346, E348, T349 (VEGF_165_ binding);R359, K373 (Heparin binding); b2 :R513, K514, K516 (Heparin binding)	VEGF family and related growth factors:VEGF-121, VEGF-B167, VEGF-C, VEGF-D, VEGF-E, VEGF_165_, VEGFR, PlGF-2, TGF-β1 and LAP, PDGF and PDGFR, PlGF-2, HGF and cMet, FGF-(1, 2, 4, 7), FGFR-1; SEMA3A(synergistic binding); SEMA3F; SEMA3G; Tuftsin (and its analogs TKPPR, A7R); miRNA/AGO2 complex; Heparin	(34, 35, 41, 51)
	C-end (MAM)	No specific amino acid position	No direct binding ligands	(33, 34)
Transmembrane domain	GxxxGxxxG Motif	G868, G876	No direct binding ligands	(34, 35)
Intracellular domain	C-end SEA motif	SEA-COOH (C-end 3 amino acids)	No direct binding ligands, Binds to proteins containing PDZ domains (NIP/GIPC, Synectin)	(34, 35, 41)

The extracellular domain is the key functional region, consisting of three subdomains: the N-terminal complement-binding-like domain A (CUB, a1/a2), the coagulation factor V/VIII homology domain B (b1/b2), and the C-terminal MAM domain (33, 34). The a1/a2 subdomains contain key amino acid positions such as H46, S47, S51, E52, K53, D78, and R80, which bind to SEMA family ligands (e.g., SEMA3A, SEMA4A) and Plexin family proteins (e.g., PlexinA1, PlexinD1). This binding activates signaling pathways including PI3K-AKT, Rho GTPase, and STAT3/STAT5, regulating immune cell migration, maintaining Treg stability, and mediating immune suppression (34–40). The b1/b2 subdomains harbor vascular endothelial growth factor (VEGF) binding positions (S346, E348, T349) and heparin binding positions (R359, K373, R513, K514, K516), which bind to growth factors such as VEGF (e.g., VEGF165), hepatocyte growth factor (HGF), and platelet-derived growth factor (PDGF). For instance, VEGF165 acts as a “bridging molecule” to strengthen the formation of the VEGFR2-NRP1 complex, significantly enhancing pro-angiogenic signals, promoting endothelial cell proliferation and migration, and inhibiting apoptosis (34, 35, 41–45). The MAM domain is mainly involved in receptor oligomerization, providing a structural basis for the formation of signal complexes between NRP1 and receptors such as VEGFR2, TGF-β1 receptor, cMet (HGFR), and PDGFR, thereby promoting EMT and CSC maintenance (33–35, 42, 46).

The transmembrane domain contains a GxxxGxxxG motif (GVLLGAVCG) with approximately 25 amino acid residues, which is a characteristic sequence distinguishing NRP1 from NRP2 (34). Key amino acid positions G868 and G876 in this motif are crucial for mediating receptor dimerization and ensuring SEMA3A signal transduction (34, 35). The intracellular domain consists of approximately 43–44 amino acid residues, with a core SEA motif (Ser-Glu-Ala-COOH) that interacts with PDZ-domain proteins (e.g., NIP/GIPC, Synectin) to mediate endothelial cell migration and downstream signal adaptation (34, 35, 41).

NRP1 lacks intrinsic enzymatic activity and functions by modulating receptor-complex assembly, endocytic trafficking, and downstream signaling. Its activity depends on ligand binding by distinct extracellular and intracellular domains. The a1/a2 domains bind SEMA family ligands implicated in immunosuppression and thus serve as the primary module for shaping the tumor immune microenvironment. The b1/b2 domains bind VEGF family ligands and thereby drive pro-angiogenic activity in tumors. The intracellular SEA motif interacts with PDZ-domain proteins to regulate downstream signal transduction. The coordinated action of these domains positions NRP1 as a central signaling hub in the tumor microenvironment.

NRP1 modulates the expression and endocytosis of VEGFR2/KDR on the cell surface to affect signal persistence and cell migration (47, 48). NRP1 forms a complex with integrin α5β1 that regulates cell polarization, ECM remodeling, angiogenesis and tumor metastasis (49); it can also negatively regulate vascular inflammation by tumor necrosis factor Alpha (TNF-α) and inhibit expression of ICAM-1 and VCAM-1, controlling inflammation in ECs (50). Functional diversity of NRP1 is reflected in its ability to bind a wide range of ligands, including members of the SEMA and VEGF families, as well as various growth factors. SEMA3A binds via the a1/a2 and b1 domains, suppressing CD8^+^ T cell activity and promoting their exhaustion. SEMA4A interacts through the a1/a2 domain to maintain Treg stability, thereby enhancing immune suppression. Members of the VEGF family (e.g., VEGF165, PlGF-2) and growth factors such as HGF and TGF-β bind through the b1/b2 domains, driving angiogenesis, tumor progression, tissue repair, and fibrosis, respectively (34, 35, 41, 51). Other ligands, such as tuftsin analogs, inhibit VEGF signaling through the b1/b2 domain to exert antitumor effects; the extracellular miRNA/argonaute 2 (miRNA/AGO2) complex mediates gene silencing through the b1/b2 domain; heparin acts as a cofactor to enhance ligand binding (34, 35, 41, 51). In melanoma, the binding of galectin-1 to NRP1 can lead to a downregulation of p27 protein expression, which in turn upregulates the epidermal growth factor receptor (EGFR) signaling pathway, resulting in adaptive resistance of tumors to BRAF inhibitors (52). Notably, NRP1 can also bind and internalize cell-penetrating peptides with a C-terminal rule (e.g., RXXR motif), thereby improving the delivery efficiency of antitumor drugs (53, 54). Its expression in endothelial cells and the regulation of the VEGF signaling pathway also exhibit organ specificity (55).

### 2.2 Expression and significance of NRP1 in tumor

#### 2.2.1 Pan-cancer expression characteristics of NRP1 in bioinformatics

**Figure 2:**
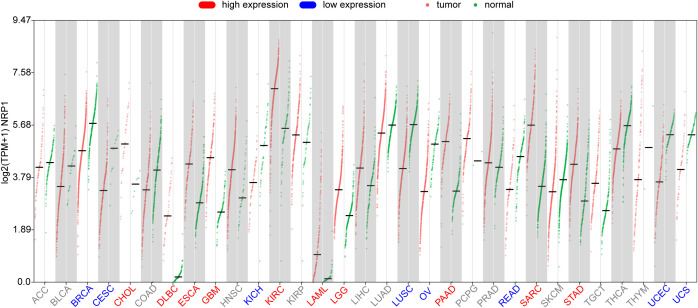
Pan-cancer expression analysis of NRP1 across TCGA and GTEx datasets. The horizontal axis represents TCGA cancer types (red dots) and corresponding normal tissues in GTEx (green dots); red fonts indicate high expression, blue fonts indicate low expression. The vertical axis represents NRP1 expression levels (log2(TPM + 1)). NRP1 is significantly differentially expressed (|log2 fold change| ≥ 1 and q-value < 0.05) in 18 out of 31 TCGA cancer types.

**Figure 3:**
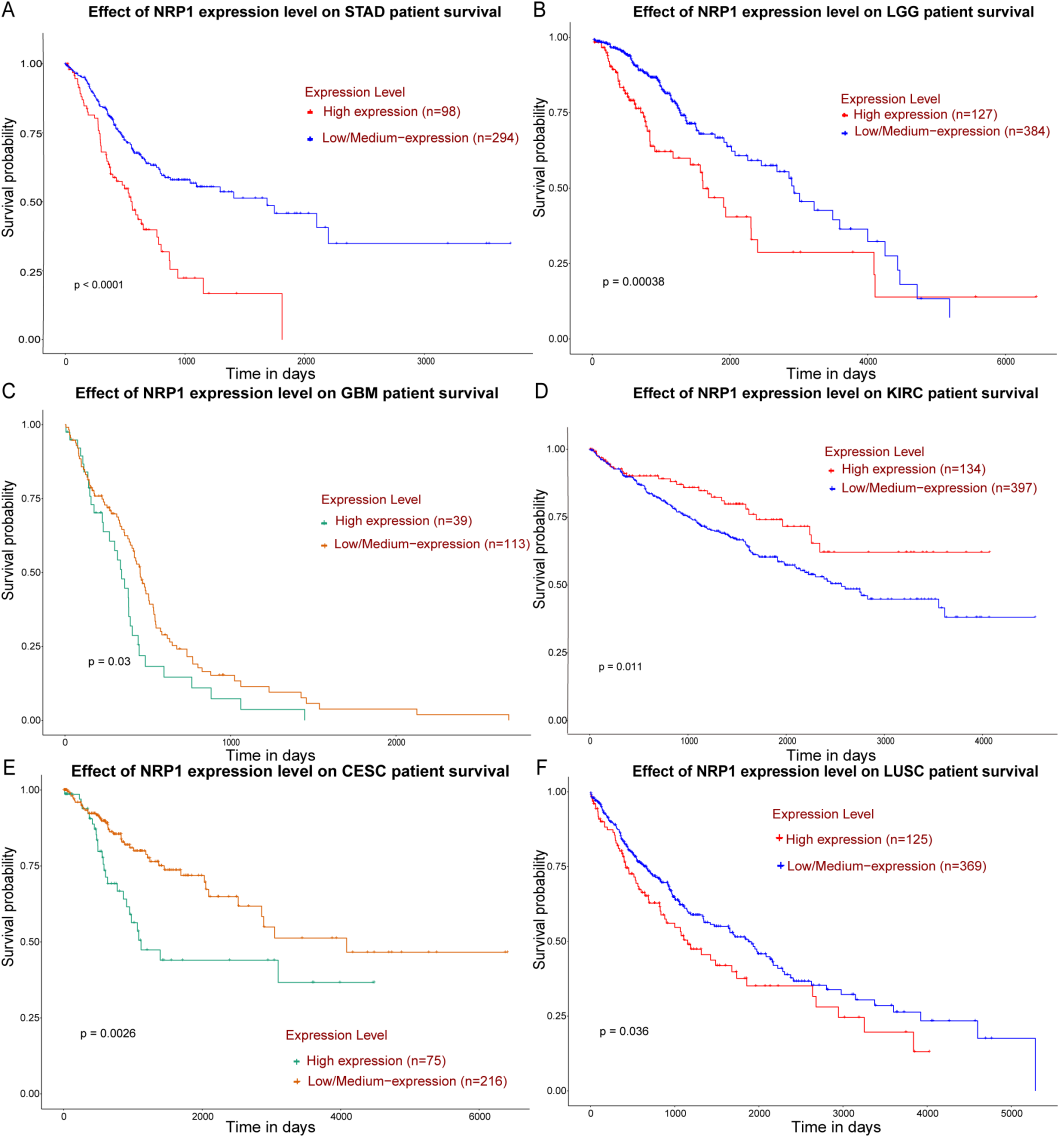
**(A–F)** Kaplan-Meier survival plots for six representative cancer types with significant NRP1 differential expression, generated using the UALCAN platform. **(A–C)** NRP1 is highly expressed in STAD, LGG, and GBM, significantly correlating with poor prognosis (p < 0.05); **(D)** NRP1 is highly expressed in KIRC but correlates with good prognosis (p < 0.05); **(E, F)** NRP1 is lowly expressed in CESC and LUSC, but high expression significantly correlates with poor prognosis (p < 0.05).

NRP1 is overexpressed in the vast majority of solid malignant tumors, and its expression pattern and prognostic value vary across cancer types. Bioinformatics analysis using the GEPIA3 platform shows that NRP1 is significantly differentially expressed in 18 of 31 TCGA cancer types (|log2 FC| ≥ 1 and q-value < 0.05; all analyses were corrected for multiple testing using FDR throughout). Lowly expressed genes with median TPM < 0.5 were filtered out (low-expression genes produce high noise and can bias correlation analyses, in accordance with standard TCGA quality-control criteria), and the expression characteristics of NRP1 were further analyzed. Compared with normal tissues from the GTEx database, NRP1 is significantly highly expressed in stomach adenocarcinoma (STAD), pancreatic adenocarcinoma (PAAD), cholangiocarcinoma (CHOL), esophageal cancer (ESCA), low-grade glioma (LGG), glioblastoma (GBM), clear cell renal cell carcinoma (ccRCC), bladder cancer (BLCA), acute myeloid leukemia (LAML), and sarcoma (SARC) (**Figure 2**). The above conclusions were also confirmed by the UALCAN public database. Survival analysis using GEPIA3 and UALCAN data platforms reveals that high NRP1 expression is significantly associated with poor prognosis in STAD (p=0.03), LGG (p=0.00038), and GBM (p<0.0001) (**Figures 3A–C**). Although NRP1 is highly expressed in ccRCC (KIRC), survival analysis shows a correlation with good prognosis (p=0.0026) (**Figure 3D**). In contrast, NRP1 is significantly lowly expressed in cervical squamous cell carcinoma (CESC) and lung squamous cell carcinoma (LUSC), but NRP1 overexpression still predicts poor prognosis in these cancers (p=0.036 for CESC, p=0.011 for LUSC) (**Figures 3E, F**), suggesting that NRP1 may exert pro-tumor effects regardless of its baseline expression level in certain contexts.

Bioinformatic analyses show that NRP1 upregulation is not random but concentrated in highly invasive, metastatic, angiogenesis-dependent tumors with cancer stem cell-like features. NRP1 is consistently overexpressed in digestive system cancers (GC, ESCA, PC, CHOL) and in nervous system tumors (CHOL, GBM). In most of these cancers, its overexpression correlates with malignant phenotypes and poor prognosis, with the exception of ccRCC, indicating that NRP1 is a common, high-frequency molecular driver of malignant progression in these tumor types.

#### 2.2.2 Biological function and prognostic value of NRP1 in cancers

Multiple studies have confirmed that NRP1 overexpression correlates with poor prognosis. In hepatocellular carcinoma (HCC), NRP1 overexpression sustains CSCs traits, drives EMT, and remodels the vasculature, fueling metastasis and predicting advanced stage and poor prognosis (8, 32, 56). Zhang et al. (9) demonstrated through public database analysis that NRP1 is highly expressed in prostate cancer (PCa) (p<0.05), and elevated NRP1 levels correlate with poorer disease-free survival (DFS) in the TCGA-PRAD cohort (p=0.0011). In GC, NRP1 interacts with FN-1 to promote EMT, accelerating tumor invasion and metastasis, and targeted blockade of this binding can inhibit tumor progression (57). In intrahepatic cholangiocarcinoma (ICC), NRP1 expression is closely correlated with tumor cell count (p=0.047), and its overexpression promotes ICC cell proliferation and migration through the FAK/PI3K/AKT pathway (11). In GBM, NRP1 overexpression is associated with CSCs maintenance and enhanced invasiveness; targeted inhibition of NRP1 reduces CSCs marker expression, improves sensitivity to temozolomide (TMZ), and significantly prolongs overall survival (OS) in patient-derived models (58, 59). In breast cancer (BC), especially triple-negative breast cancer (TNBC) and the claudin-low subtype, NRP1 overexpression is linked to CSC maintenance, RAS/MAPK pathway activation, and increased invasiveness, with patients showing significantly shortened DFS and OS; targeted inhibition of NRP1 can effectively inhibit tumor progression (60, 61). Rachner et al. (62) detected serum NRP1 levels of 509 early-stage BC patients at the initial diagnosis and found that soluble NRP1 levels are negatively correlated with OS. In head and neck squamous cell carcinoma (HNSCC), NRP1 overexpression promotes Treg and M2-like macrophage infiltration, inhibits immune effector cell function, forms STIME, and leads to tumor immune escape and poor prognosis (14). In lung adenocarcinoma (LUAD), HIF-1α directly binds to the NRP1 promoter to regulate its expression; NRP1 overexpression is a core driver of tumor metastasis and vasculogenic mimicry formation, serving as a prognostic marker and potential therapeutic target (10). In colorectal cancer (CRC), NRP1 activates the cytoskeleton regulatory protein Cdc42 through the VEGF pathway to promote migration and invasion, with overexpression associated with poor prognosis (63). In pancreatic cancer (PC), NRP1 overexpression correlates with increased angiogenesis, lymph node metastasis, and advanced clinical staging, leading to reduced OS (64–66); however, MORIN et al. (67) found that NRP1 overexpression in PC can form a VEGFR2/NRP1 transcomplex, reducing tumor vascular area and inhibiting growth, improving OS, highlighting its functional heterogeneity. In medulloblastoma (MB), the relationship between NRP1 expression and prognosis is complex, with low NRP1 expression being associated with poorer survival (68). In ccRCC, NRP1 exerts bidirectional regulatory effects: promoting tumor fibrosis and EMT via the TGF-β/SMAD pathway while inhibiting apoptosis and facilitating cell cycle progression, but also enriching in the cellular stroma to form a barrier against tumor progression, correlating with favorable prognosis (12). It has been shown that NRP1 regulates tumor angiogenesis and TME remodeling by binding to multiple ligands such as the VEGF family and TGF-β1, and promote tumor growth and metastases (7, 16).

Drug resistance is a major cause of tumor treatment failure, and NRP1 overexpression is a key factor mediating resistance to various therapies. In bladder cancer, NRP1 is related to remodel the STIME, predict the response to immune checkpoint inhibitors (ICIs) and cisplatin adjuvant chemotherapy (15). Rizzolio et al. (69) found NRP1 overexpression in BRAF inhibitor-resistant melanoma and HER2-targeted therapy-resistant BC cells, identifying NRP1 as a novel therapeutic target for drug resistance. In CRC, NRP1 expression is significantly higher in tumor tissues than in normal tissues, and high NRP1 expression in T4-stage tumors is associated with significantly shortened OS (p=0.024); NRP1 overexpression is positively correlated with Irinotecan resistance, serving as a key mediator of chemoresistance (31). In ovarian cancer (OC), NRP1 overexpression correlates with Olaparib resistance; SKOV3 cells overexpressing NRP1 are naturally resistant to Olaparib, while NRP1 silencing restores sensitivity (36% decrease in cell viability, reduced colony-forming ability, and activation of the apoptosis pathway) (70). Additionally, NRP1 maintains the characteristics and invasiveness of CSCs by forming complexes with VEGF and GIPC1, respectively, and is closely correlated with drug resistance and tumor recurrence (7, 32). These findings confirm that NRP1 is not only a key regulator of tumor progression but also a prognostic biomarker and therapeutic target with broad application potential.

### 2.3 NRP1 multiple roles in TME

**Figure 4:**
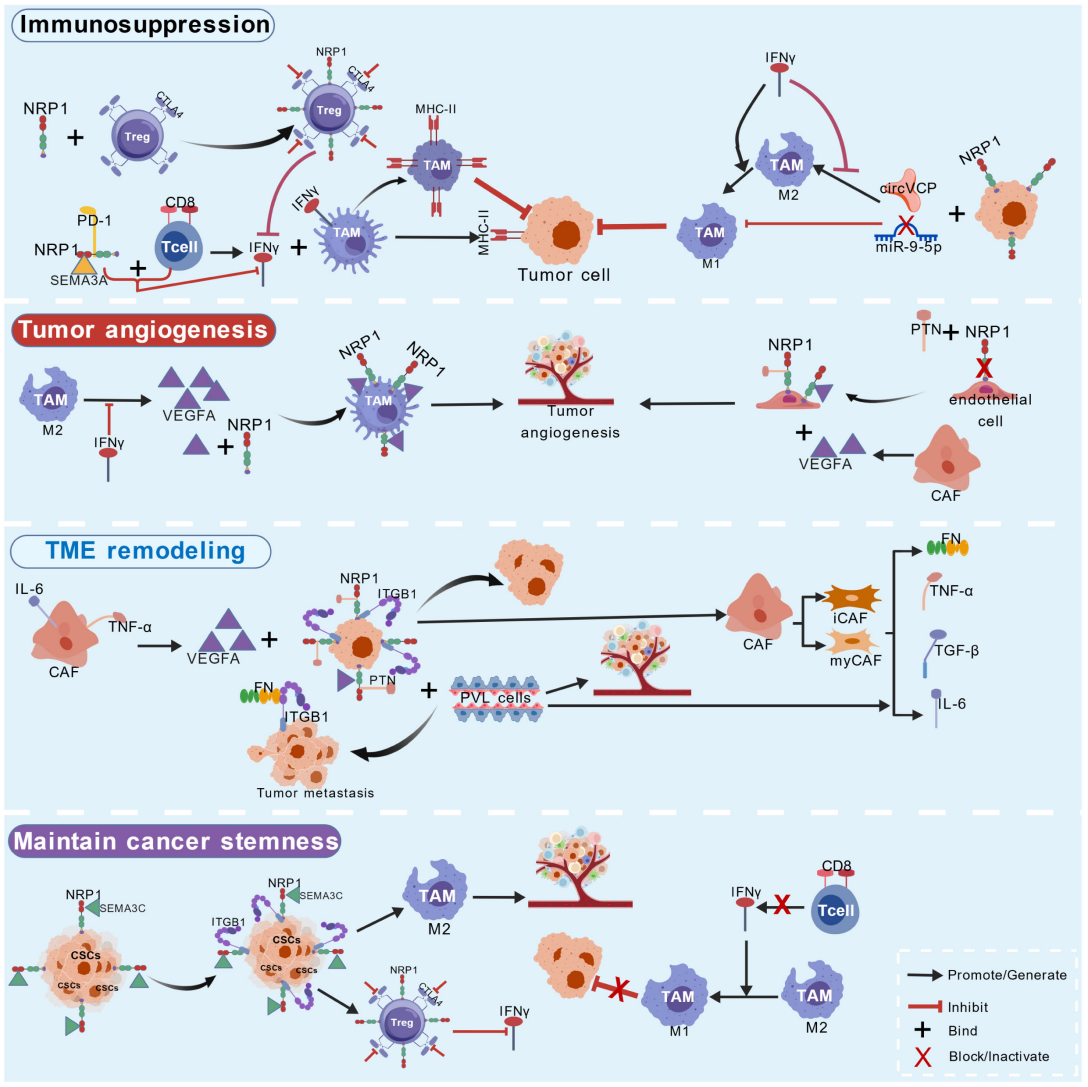
NRP1 plays diverse roles in the TME. Immunosuppression: NRP1^+^ Tregs suppress IFN-γ secretion via CTLA4 inhibition, sustaining M2-like macrophage activity. NRP1 on CD8^+^ T cells binds SEMA3A to block IFN-γ-driven MHCII^high^ antitumor macrophage differentiation, synergizing with PD1 to amplify immune suppression. The miR-9-5p/NRP1 axis further polarizes macrophages toward M2. Tumor angiogenesis: M2-derived VEGFA binds NRP1 on TAMs to promote vessel formation. PTX activates NRP1 on tumor endothelial cells (TECs), stimulating endothelial proliferation and tip cell enrichment; recruited CAFs secrete additional VEGFA, forming a proangiogenic feedback loop. TME remodeling: NRP1-ITGB1 signaling between CAFs and tumor cells upregulates EMT and inflammation-related genes, enhancing invasiveness. NRP1 drives CAFs toward an iCAF/myCAF double-positive state, secreting ECM components and inflammatory mediators to reshape the stromal niche. Maintain cancer stemness: Tumor-secreted SEMA3C interacts with NRP1/ITGB1, activating stemness-associated pathways to sustain CSC self-renewal. CSCs release TGF-β and VEGFA, reinforcing M2 polarization and Treg expansion while inhibiting CD8^+^ T cells, perpetuating an immunosuppressive, CSC-supportive microenvironment. Created with BioGDP.com (158).

NRP1 acts as a crucial signaling hub in the TME, directly regulating immune suppression, angiogenesis, TME remodeling, and cancer cell stemness through dynamic interactions with immune cells, ECs, and CSCs. Its dysregulation correlates with poor prognosis and therapeutic resistance in various cancers. NRP1 overexpression positively correlates with the infiltration of immunosuppressive cells (CAFs, Tregs, M2-like macrophages) and cytotoxic CD8^+^ T cell, contributing to STIME formation (13, 14, 71) (**Figure 4**).

#### 2.3.1 NRP1 in immune suppression and immune escape

NRP1 drives STIME formation to promote tumor growth and immune escape (72). NRP1^+^ Tregs are enriched in various malignancies and play a role in tumor immune escape, and their abundance is associated with poor prognosis (73, 74). Overexpression of NRP1 on intratumoral Tregs enhances their stability, anti-apoptotic ability, and proliferative activity, boosting immunosuppressive function. Blocking NRP1 reduces Treg suppression, and MAPK pathway inhibitors can downregulate NRP1 expression. In multiple cancers (including HNSCC), increased NRP1^+^ Tregs correlate with shorter progression-free survival (PFS), and their expression in peripheral blood may be associated with poor prognosis (73). NRP1^+^ Tregs suppress the function of cytotoxic T lymphocyte associated antigen 4 (CTLA-4) and inhibit interferon-γ (IFN-γ) secretion, indirectly supporting M2-like macrophage metabolic adaptation, maintaining mitochondrial integrity, and increasing survival (35, 38, 74, 75). NRP1 overexpression is also associated with increased Treg and M2-like macrophage numbers, accelerating tumor progression and driving immune escape (13, 14, 16).

NRP1 is highly expressed on activated CD8^+^ T cells, where it cooperates with programmed cell death receptor 1 (PD-1) to enhance PD-1-mediated inhibition, reducing ICIs efficacy. In melanoma models, selective knockdown of NRP1 in CD8^+^ T cells restores antitumor activity and potentiates anti-PD-1 therapy. Clinically, overexpression of NRP1 on the surface of tumor-infiltrating CD8^+^ T cells strongly correlates with anti-PD-1 resistance and reduced relapse-free survival in melanoma patients (76). Overexpression of NRP1 in TAMs enhances tumor progression by changing the TME and angiogenesis, which are closely associated with immune evasion and poor survival. In CRC, tumor cells regulate macrophage polarization between M1 and M2 states via the miR-9-5p/NRP1 axis, which modulates exosomal circVCP secretion, leading to tumor growth and metastasis. Nanobodies targeting NRP1 can disrupt its interactions and shift the TME towards a proinflammatory state, potentially increasing antitumor immune response (77). Notably, targeting NRP1 can reverse immune suppression. Nanobody blockade of NRP1 and SEMA3A interaction allows accumulation of proinflammatory MHC-II^high^ macrophages, tumor specific CD8^+^ T cell infiltration and inflamed TME, which inhibit tumor growth and prolong OS (78). Notably, NRP1 expressed on CD8^+^ T cells synergizes with PD-1 to suppress T cell effector functions, thereby contributing to the conversion of hot tumors into cold tumors and promoting resistance to immune checkpoint inhibitors (76). Thus, targeting NRP1 on T cells may represent a strategy to reinvigorate antitumor immunity and sensitize cold tumors to immunotherapy.

#### 2.3.2 NRP1 in tumor angiogenesis

NRP1 is involved in intercellular signal transduction, leading to abnormal vascular remodeling and dysregulation. In pancreatic ductal adenocarcinoma (PDAC), co-culture of CAFs with tumor cells markedly increases VEGFA secretion; CAF-derived VEGFA binds NRP1 and activates downstream cascades that promote endothelial proliferation and angiogenesis. Functional studies demonstrated that the conditioned medium from these co-cultures promotes network formation in human umbilical vein endothelial cells (HUVECs), while NRP1 knockdown attenuates this effect (79). NRP1 directly regulates tumor angiogenesis. Pleiotrophin activates NRP1 in ECs, enriching tip cells and activating sprouting pathways, which promote angiogenesis and metastatic potential (80). A study on MB found persistent NRP1 overexpression in tumor vascular ECs and TAMs, suggesting coordinated regulation of angiogenesis and immune modulation by these cell populations (81).

#### 2.3.3 NRP1 in TME remodeling and cancer stemness maintenance

NRP1 fosters a pro-invasive TME by mediating interactions between CAFs and tumor cells. In PDAC, CAFs engage tumor cells through integrin beta 1 (ITGB1) signaling, while NRP1, as a VEGFA co-receptor, contributes to the CAFs-tumor cell crosstalk and cooperatively upregulates EMT and inflammation-related genes (e.g., IFN-γ response genes). This gene program shifts tumor cells toward a plastic state that exhibits mixed basal/classical subtype features and increased invasiveness. Meanwhile, CAFs and tumor cell co-culture induce CAFs phenotypic conversion, expanding the iCAF/myCAF double-positive population, which further remodels the TME by secreting ECM components and inflammatory factors (e.g., TNF-α, IL-6, TGF-β) (79). In HCC, tumor-secreted SEMA3C engages NRP1 and ITGB1 to activate the AKT/Gli1/c-Myc axis, which enhances CSC self-renewal, stimulates hepatic stellate cell activation, and remodels the extracellular matrix, thereby promoting formation of the STIME (82). In inflammatory breast cancer (IBC), NRP1-driven angiogenesis recruits immature perivascular cells (PVL cells, e.g., MCAM^+^ CD36^+^ subtype) that accumulate in affected skin. These cells create a pro-metastatic niche by secreting ECM proteins (e.g., FN-1) and inflammatory mediators, and they engage endothelial cells through the PDGFβ-PDGFRβ axis to stabilize nascent vessels and increase vascular permeability, facilitating tumor cell migration and distant metastasis (80).

Due to its multiple roles in regulating interactions between neoplastic and non-neoplastic components in the TME, targeting NRP1 can both inhibit tumor growth and metastasis and enhance immunotherapy efficacy, indicating substantial potential for clinical translation (7, 83–85).

## 3 The role and regulatory mechanism of autophagy in tumors

### 3.1 Core mechanisms and dual roles of autophagy in tumorigenesis

**Figure 5:**
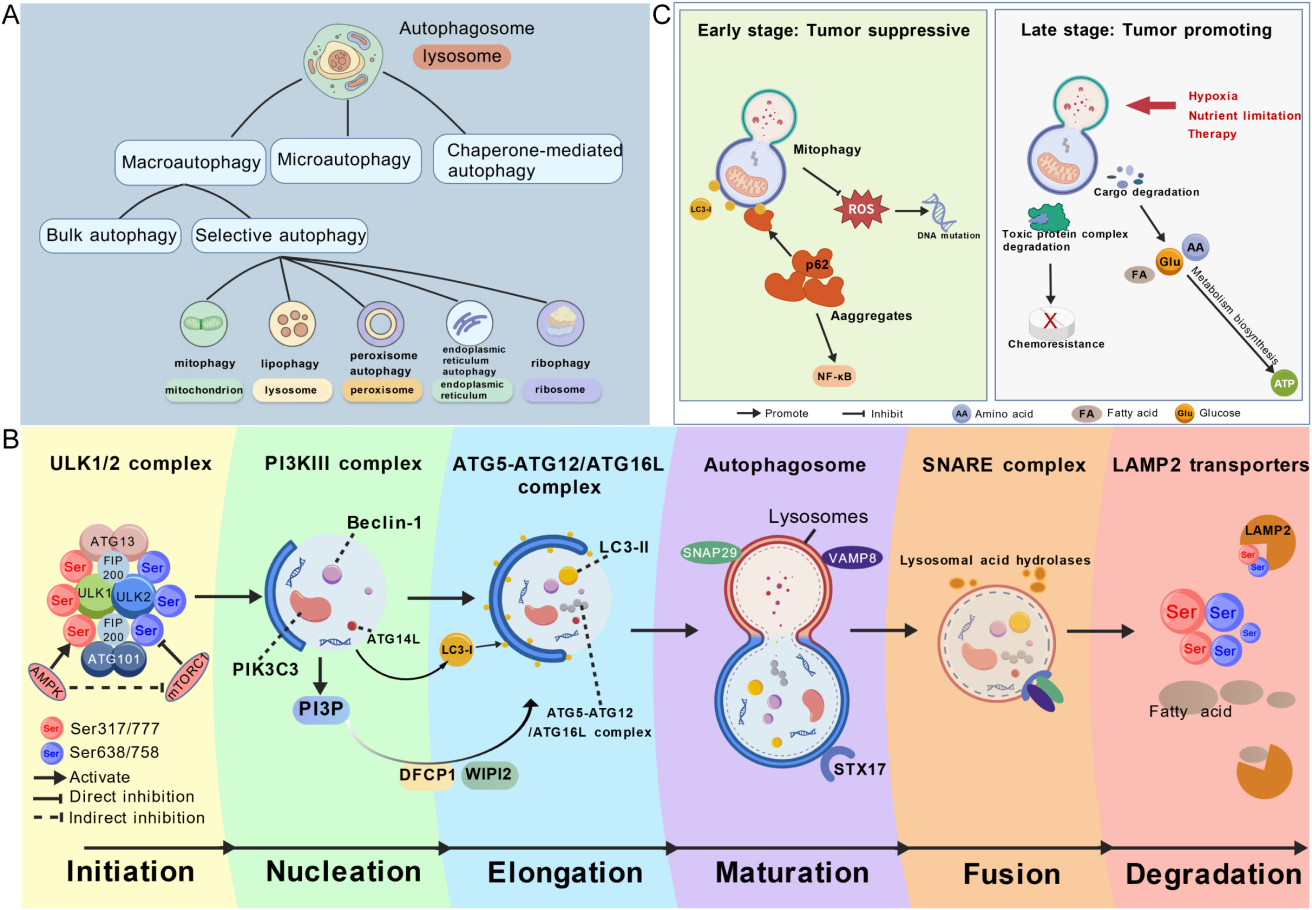
**(A)** Classification of autophagy. **(B)** Molecular mechanisms of autophagy: The ULK1 complex (regulated by AMPK and mTORC1) initiates autophagy. During nucleation, the PI3KIII complex generates PI3P, recruiting WIPI2 and DFCP1. In elongation, the ATG5-ATG12/ATG16L complex facilitates LC3 esterification. Maturation forms closed autophagosomes, which fuse with lysosomes via the SNARE complex (STX17, VAMP8, SNAP29). Lysosomal acid hydrolases degrade cargos, with products recycled via LAMP2 transporters. **(C)** Dual role of autophagy in tumor progression: Early stage autophagy suppresses tumors by reducing ROS and inhibiting NF-κB activation; late stage autophagy is hijacked by tumors to support growth and chemoresistance. Created with BioGDP.com (158).

Autophagy is a highly conserved cellular self-degradation process. Through autophagosomes, cells sequester cytoplasmic components such as damaged proteins, lipids, and organelles within the cell, which ultimately fuse with lysosomes for degradation and recycling. Autophagy mainly includes three types: macroautophagy, chaperone-mediated autophagy, and microautophagy (**Figure 5A**). Although the three types vary morphologically, they all ultimately deliver cargos to lysosomes for degradation and recycling (86). Usually, the autophagy we discuss refers to macroautophagy (86). Macroautophagy includes basal autophagy and selective autophagy, and the latter is further divided into mitophagy, lipophagy, pexophagy, reticulophagy, and ribophagy (87).

The regulatory mechanism of autophagy involves a series of ATGs and their encoded protein complexes, including the unc-51-like kinase 1/2 (ULK1/2) complex, class III phosphatidylinositol-3-phosphate (PI3P) complex, ATG9A transport system, and ATG12-LC3 ubiquitin-like conjugation system. These factors participate in the initiation, nucleation, elongation, maturation, fusion, and degradation stages of autophagy (**Figure 5B**). In the initiation stage, cellular stresses such as nutrient deficiency and abnormal energy metabolism can activate the ULK1 complex (ULK1/ATG13/FIP200/ATG101) by inhibiting mTOR activity or activating AMPK, which directly phosphorylates ULK1 (at Ser317 and Ser777 positions) and indirectly inhibits mTOR, initiating autophagy (88–90). Entering the nucleation stage, the ULK1 complex phosphorylates ATG13 and ATG14L, activating the class III PI3K kinase complex (Beclin1/PIK3C3 [VPS34]), which mediates PI3P production to recruit WIPI2 and DFCP1, forming an autophagosome nucleation platform (23, 24, 26). In the elongation stage, the ATG5-ATG12-ATG16L complex localizes to the nucleation platform, initiating lipidation of LC3 (ATG8 family protein). LC3-I binds phosphatidylethanolamine (PE) to generate LC3-II, which inserts into the autophagosome membrane, promoting membrane elongation and enclosing cargos (26, 90, 91). Subsequently, the autophagosome membrane closes, forming a mature double-membrane structure marked by LC3-II, completing autophagosome maturation. The mature autophagosome fuses with the lysosome membrane through the SNARE complex formed by STX17 on its surface and VAMP8 and SNAP29 on the lysosome, forming an autolysosome (26, 90, 91). Finally, in the degradation stage, the lysosomal acid hydrolases degrade cargos into small molecules (amino acids, fatty acids), which are released into the cytoplasm via LAMP2-related transporters for recycling (26, 90, 91).

Autophagic activity is elevated in various malignant tumors, regulating the growth and apoptosis of tumor cells and being associated with the malignancy of tumors. Both activation and inhibition of autophagy can enhance antitumor drug efficacy, but upregulation of autophagy during treatment may induce drug resistance and correlate with poor prognosis (21, 26). In GC and OC, risk models based on ATGs or long non-coding RNA (lncRNA) can predict prognosis and ICIs response (92, 93). Approximately 3%–32% of ATGs are significantly differentially expressed in 21 human cancer types, and autophagy-related scores constructed from these ATGs correlate with drug sensitivity, highlighting their value in cancer treatment and prognosis (94). In CRC research, integrating immune-related genes with ATGs identified 22 immune-autophagy-related genes (IATGs) correlated with low-risk CRC, and targeting these genes may reduce clinical risk (95).

Autophagy exhibits a stage-dependent dual role in tumorigenesis and progression (**Figure 5C**). In the early stage of tumorigenesis, autophagy reduces the generation of reactive oxygen species (ROS) via mitophagy, lowering the risk of DNA mutations (24). Meanwhile, selective autophagy mediated by SQSTM1/p62 efficiently clears abnormal cargos, preventing activation of pro-oncogenic signaling pathways such as NF-κB (23). When hepatocyte autophagy is defective, it leads to the p62 aggregates, inducing chronic inflammation and the occurrence of HCC (96). In tumor progression or the advanced stage, tumor cells hijack autophagy to adapt to stress conditions (hypoxia, nutrient deprivation). Autophagy recycles nutrients (amino acids, lipids) to provide energy and raw materials for biosynthesis, interacts with EMT-related signaling pathways (e.g., SNAI1, ZEB1/2, NOTCH1), regulates glucose and glutamine metabolic reprogramming, and supports tumor proliferation, invasion, and metastasis, showing a pro-cancer effect (17–26, 97). In a PC model, autophagy deficiency prevents utilization of autophagy-derived glutamine for mitochondrial metabolism, significantly inhibiting KRAS-driven tumor growth (90). Additionally, autophagy regulates core pathways (e.g., PI3K-AKT-mTOR, AMPK) by selectively degrading cell cycle regulatory proteins and cancer-related signaling molecules, participating in genome repair, cell cycle control, and CSC homeostasis maintenance, laying the foundation for tumor recurrence and metastasis (22, 23). Autophagy can also degrade toxic protein complexes induced by chemotherapy drugs (e.g., DNA complexes formed by topoisomerase inhibitors), leading to chemoresistance (98). This dual role determines that autophagy-targeted therapeutic strategies (activation or inhibition) must be tailored to tumor type and development stage, with reasonable regulation of autophagy to avoid drug resistance and ensure optimal antitumor efficacy.

### 3.2 Key signaling pathways regulating autophagy in tumors

**Figure 6:**
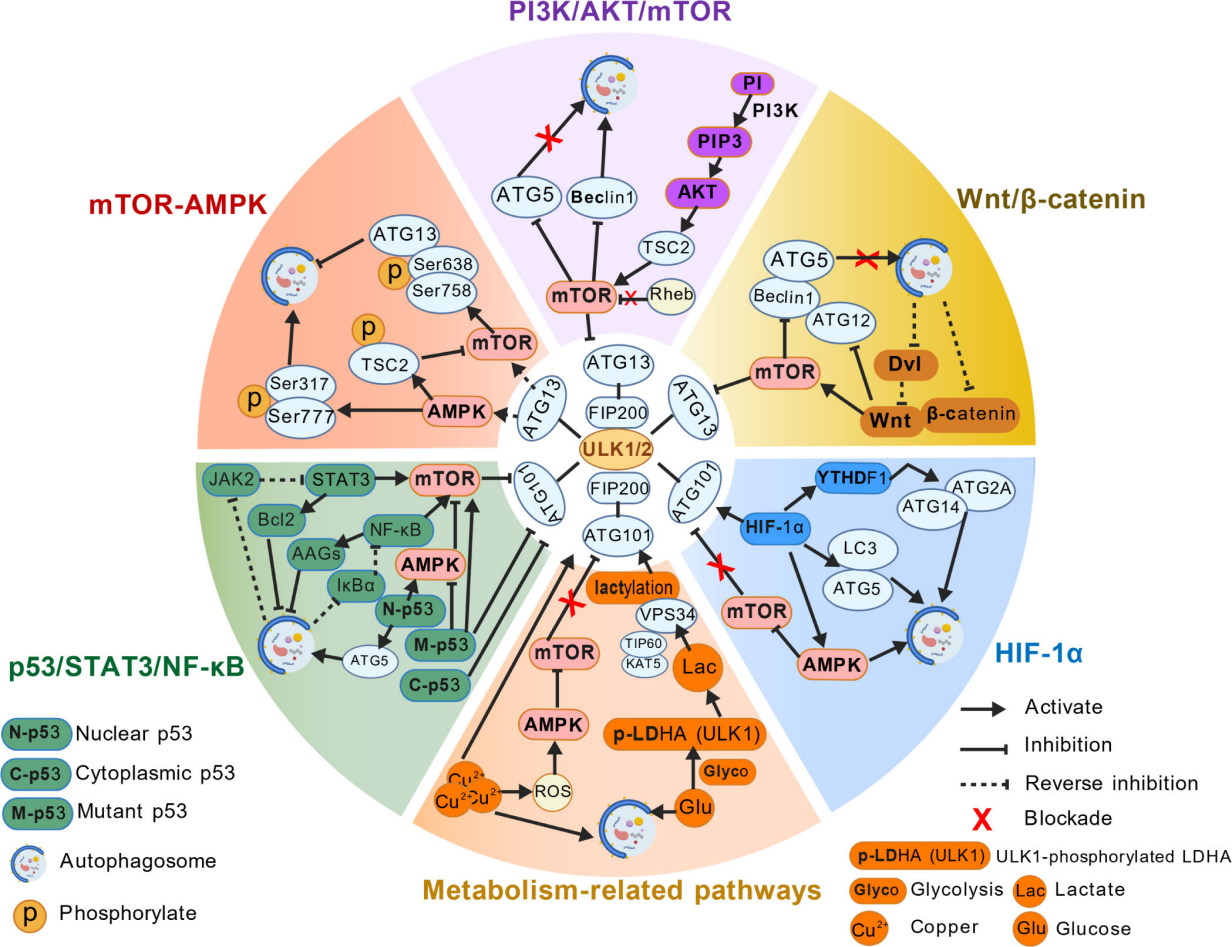
Autophagy-related signaling pathways in cancer. Multiple pathways (mTOR-AMPK, PI3K/AKT/mTOR, Wnt/β-catenin, HIF-1α, p53/NF-κB/STAT3, metabolism-related) coordinately regulate autophagy, integrating signals from the TME to influence tumor progression and treatment sensitivity. Created with BioGDP.com (158).

Autophagy is strictly regulated by a coordinated multi-pathway network centered on mTOR and AMPK, integrating growth signals, hypoxia responses, and metabolic signals to determine the autophagic phenotype (protective or pathogenic) of tumor cells, ultimately influencing tumor progression and treatment sensitivity (**Figure 6**).

#### 3.2.1 mTOR-AMPK signaling pathway

As the “dual sensors” of cellular energy and nutritional status, mTOR and AMPK bidirectionally regulate autophagy initiation through competitive phosphorylation of the ULK1 complex (23). mTOR is the main negative regulator of autophagy. In nutrient-sufficient conditions, mTORC1 phosphorylates ULK1 (Ser638/Ser758), ATG13 (Ser258), and the transcription factor TFEB, which directly inhibits the autophagy initiation complex and lysosome biogenesis. Targeted inhibition of mTOR (e.g., rapamycin analogs) relieves this suppression (90). Under energy depletion or hypoxia in the TME, liver kinase B1 (LKB1) activates AMPK, which directly phosphorylates ULK1 (Ser317/Ser555) or indirectly inhibits mTORC1 via TSC2 phosphorylation (Ser1387), doubly activating autophagy. The negative-feedback loop formed by the two enables tumor cells to dynamically adjust the autophagy level according to the energy fluctuations in the microenvironment. This mechanism is particularly significant in PC and CRC (23, 90).

#### 3.2.2 PI3K/AKT/mTOR signaling pathway

The PI3K/AKT/mTOR pathway is a crucial signaling axis for tumorigenesis and a core negative regulatory pathway of autophagy. PI3K catalyzes phosphatidylinositol conversion to phosphatidylinositol-3,4,5-trisphosphate (PIP3), recruiting and phosphorylating AKT for activation. Activated AKT further phosphorylates TSC2 (at Ser939/Thr1462 positions), relieving its inhibition on the small G protein Rheb, ultimately activating mTORC1 (99). Abnormal activation of this pathway (e.g., PI3K mutations, AKT amplification, PTEN deletion) frequently occurs in various tumors, including BC and PCa. It comprehensively inhibits autophagy by suppressing the activity of the ULK1 complex and downregulating the expression of core ATGs (e.g., Beclin1, ATG5) (99–101). This autophagy inhibition enables tumor cells to evade metabolic stress-induced death and reduce clearance of intracellular toxic substances, promoting proliferation, invasion, and chemoresistance (99–101).

#### 3.2.3 Wnt/β-catenin signaling pathway

The interplay between the Wnt/β-catenin signaling pathway and autophagy is complex and context-dependent, demonstrating bidirectional regulation within tumor environments. In most solid tumors, abnormal pathway activation (e.g., adenomatous polyposis coli [APC] mutation, nuclear β-catenin accumulation) can inhibit autophagy by directly downregulating the transcription of core ATGs (e.g., Beclin1, ATG5, ATG12) or indirectly regulating the mTOR/AMPK signaling axis, providing a protective microenvironment for tumor proliferation (92, 102). In CRC, mutation of the APC gene leads to persistent pathway activation, suppresses ATG5 expression via the β-catenin-TCF/LEF complex, decreases autophagy levels, and enhances self-renewal and chemoresistance in CSCs (102). Autophagy exerts reverse regulation on this pathway; in GBM, autophagy can selectively degrade dishevelled (Dvl) protein or β-catenin, inhibiting pathway activity and weakening tumor stem cell stemness (102, 103). In HNSCC, Wnt3a ligand activates the pathway while upregulating the LC3-II/LC3-I ratio and autophagosome formation, enhancing radioresistance to show a synergistic activation effect (102). In GBM, P4HB promotes β-catenin nuclear translocation and inhibits autophagy by regulating Wnt3-LRP6 binding, maintaining CSC sphere-forming ability (103). In the GC model, pathway activation can downregulate LC3-II level and the number of autophagosomes, and BML-284 (a Wnt/β-catenin pathway activator) can reinforce this effect, confirming the negative regulation of the pathway in autophagy (92).

#### 3.2.4 HIF-1α signaling pathway

Hypoxia is a typical characteristic of the solid TME, and the HIF-1α-mediated hypoxia response pathway is closely related to autophagy activation. Under hypoxic conditions, HIF-1α can directly bind to the HBS1 and HBS3 sites of the promoter of the m^6^A reader YTHDF1, transcriptionally activating the expression of YTHDF1. YTHDF1 recognizes m^6^A modification sites on ATG2A and ATG14 mRNAs via its m^6^A-binding pocket (residues K395/Y397), thereby enhancing the translational efficiency of these transcripts to activate autophagy (104). In HCC, this hypoxia-induced autophagy mediated by the HIF-1α-YTHDF1-ATG2A/14 axis significantly enhances the hypoxia adaptability and metastatic ability of tumor cells, and overexpression of YTHDF1 is associated with poor prognosis in patients (104). This mechanism reveals the crucial role of m^6^A epigenetic modification in the regulation of tumor autophagy and provides new targets for targeting hypoxia-related autophagy.

#### 3.2.5 P53/NF-κB/STAT3 signaling pathways

Nuclear-localized p53 activates transcription of target genes (e.g., DRAM1, PRKAB1) under stress (DNA damage) to promote autophagy initiation, while cytoplasmic p53 directly suppresses the autophagy complex. Common p53 mutations in tumors disrupt this bidirectional regulation, and some mutant p53s enhance tumor cell proliferation by inhibiting autophagy (23). Research indicates that in hypoxic or nutrient-deprived conditions, p53 is suppressed while autophagy is activated to support cell survival. When autophagy is inhibited, however, p53 activity is restored. Through nuclear localization, p53 then activates apoptotic target genes, which ends autophagy-mediated protection and induces apoptosis in dysfunctional endothelial cells. This process ultimately results in tumor vascular remodeling and the optimization of the immune microenvironment (105).

Activated NF-κB can indirectly inhibit autophagic flux by upregulating the expression of anti-apoptotic genes (AAGs) such as Bcl-xL. Meanwhile, autophagy can relieve the inhibition of NF-κB by degrading IκBα, an inhibitor of NF-κB, promote the continuous activation of the pathway, and then participate in the formation of the tumor inflammatory microenvironment. This interaction is particularly prominent in inflammation-related tumors (e.g., colitis-associated CRC) and significantly impacts CRC, GC, and HCC progression (20, 23).

STAT3 can raise expression of anti-autophagic molecules (e.g., Bcl-2), directly inhibiting autophagy. Autophagy can selectively lower certain components of the STAT3 pathway (e.g., JAK2) and suppress the pathway activity in a negative feedback loop. It may also lead to tumor immune escape by inhibiting T cell function. Aberrant autophagy regulated by this pathway has been reported clearly in lymphoma, BC, and lung cancer (20).

#### 3.2.6 Cell metabolism-related signaling pathways

Lactate metabolism and autophagy are linked through regulatory mechanisms. ULK1 phosphorylates lactate dehydrogenase (LDHA) under nutrient-poor conditions. In addition to LDHA activity and lactate production, lactate is used by KAT5/TIP60 to lactylate PIK3C3/VPS34, which increases autophagy function. This promotes autophagosome formation and maturation, helping cells to adapt to nutrient-poor environments and tumor growth. The link between glycolysis and autophagy provides potential therapeutic targets for tumor metabolism-autophagy regulation (106). Copper ions regulate autophagy directly by activating ULK1/2 kinases and indirectly by inducing ROS production to stimulate autophagy through the AMPK-mTOR pathway. In KRAS-driven lung cancer, increased copper uptake via SLC31A1 activates autophagy, providing essential nutrients for tumor cell proliferation. Copper signaling connects TAX1BP1-mediated GPX4 degradation to ferroptosis, establishing a crucial “copper-autophagy-ferroptosis” network with clinical relevance for therapeutic response in CRC and BC (107).

The above pathways do not exist in isolation but form a regulatory network through cross-talk (**Table 2**). For example, the PI3K/AKT/mTOR pathway can enhance the stability of HIF-1α through phosphorylation, indirectly regulating hypoxia-related autophagy (104); AMPK not only regulates mTOR but can also phosphorylate β-catenin to affect the activity of the Wnt pathway (102). This complex interaction endows tumor autophagy with high plasticity and provides a theoretical basis for the development of multi-targeted combination therapy strategies.

**Table 2:** Autophagy regulation by core signaling pathways in different cancers.

Signal pathway	Cancer	Autophagy regulation	Regulatory mechanisms and key molecules	Refs
mTOR-AMPK	PC, CRC	Bidirectional (energy/nutrient status)	Nutrient sufficiency: mTORC1→ phosphorylates ULK1 (Ser 638/Ser 758), ATG13 (Ser 258), and transcription factor TFEB→ autophagy inhibition; Energy depletion: LKB1→ activates AMPK→ AMPK directly phosphorylates ULK1 (Ser 317/Ser 555) OR phosphorylates TSC2 (Ser 1387) → indirectly inhibits mTORC1→ dual autophagy activation.	(23, 90)
PI3K/AKT/mTOR	BC, PCa	Inhibition	PI3K→ phosphatidylinositol conversion to PIP3→ recruitment and phosphorylation of AKT→ AKT activation→ AKT phosphorylates TSC2 (Ser939/Thr1462)→ relief of TSC2-mediated inhibition of Rheb→ mTORC1 activation→ suppression of ULK1 complex activity + downregulation of core ATGs (e.g., Beclin1, ATG5)→ autophagy inhibition.	(99–101)
Wnt/β-catenin	GC	Inhibition	Wnt/β-catenin pathway activation → LC3-II/LC3-I ratio significantly reduced → autophagy inhibition.	(92)
	CRC	Inhibition	APC mutation → β-catenin persistent activation → inhibition of ATG5 expression in CSCs → autophagy inhibition.	(102)
	HNSCC	Activation	Wnt3a ligand activates the pathway → upregulation of LC3-II/I ratio and autophagosome formation → autophagy activation.	(102)
	GBM	Bidirectional (inhibition-dominant)	Autophagy degrades Dvl or β-catenin → pathway inhibition; P4HB promotes β-catenin nuclear entry and inhibits autophagy → maintenance of CSC stemness.	(102, 103)
HIF-1α	HCC	Activation	HIF-1α → transcriptionally activates YTHDF1 → enhances translation efficiency of ATGs (e.g., ATG2A, ATG14) → autophagy activation.	(104)
p53	BC, HCC, CRC	Bidirectional (tumor-suppressive/promoting)	Nuclear p53 → activation of autophagy-promoting genes (e.g., DRAM1) → autophagy activation; Cytoplasmic p53 → direct inhibition of autophagy complexes → autophagy inhibition; Mutant p53 → inhibition of autophagy → promotion of cell proliferation.	(23, 105)
NF-κB	BC, HCC, CRC	Inhibition (indirect)	NF-κB → upregulates Bcl-xL → autophagy flux blocked; Autophagy degrades IκBα → enhanced NF-κB activation (positive feedback).	(20, 23)
STAT3	Lymphoma, BC, Lung cancer	Inhibition	STAT3 → upregulates Bcl-2 → inhibits Beclin1 complex formation → autophagy inhibition.	(20)
Metabolic pathways (lactate/copper)	CRC, BC	Activation	ULK1 → phosphorylates LDHA → promotes lactylation → enhanced autophagic function; Copper ions → activates ULK1/2 or ROS-AMPK pathway → autophagy induction → crosstalk with ferroptosis.	(106, 107)

### 3.3 Dual roles of autophagy in the TME

The TME is a dynamic ecosystem composed of tumor cells, various non-tumor cells, extracellular matrix, cytokines, metabolites, etc. Autophagy, as a conserved substance degradation and recycling process in eukaryotic cells, occupies a central position in the TME regulatory network. Interactions between TME components influence autophagy, which in turn regulates tumor initiation, growth, metastasis, and treatment response (108). Autophagy acts as a “double-edged sword” in the TME, either inhibiting tumor growth or promoting malignancy depending on context.

#### 3.3.1 Autophagy in tumor-stromal cell interactions

**Figure 7:**
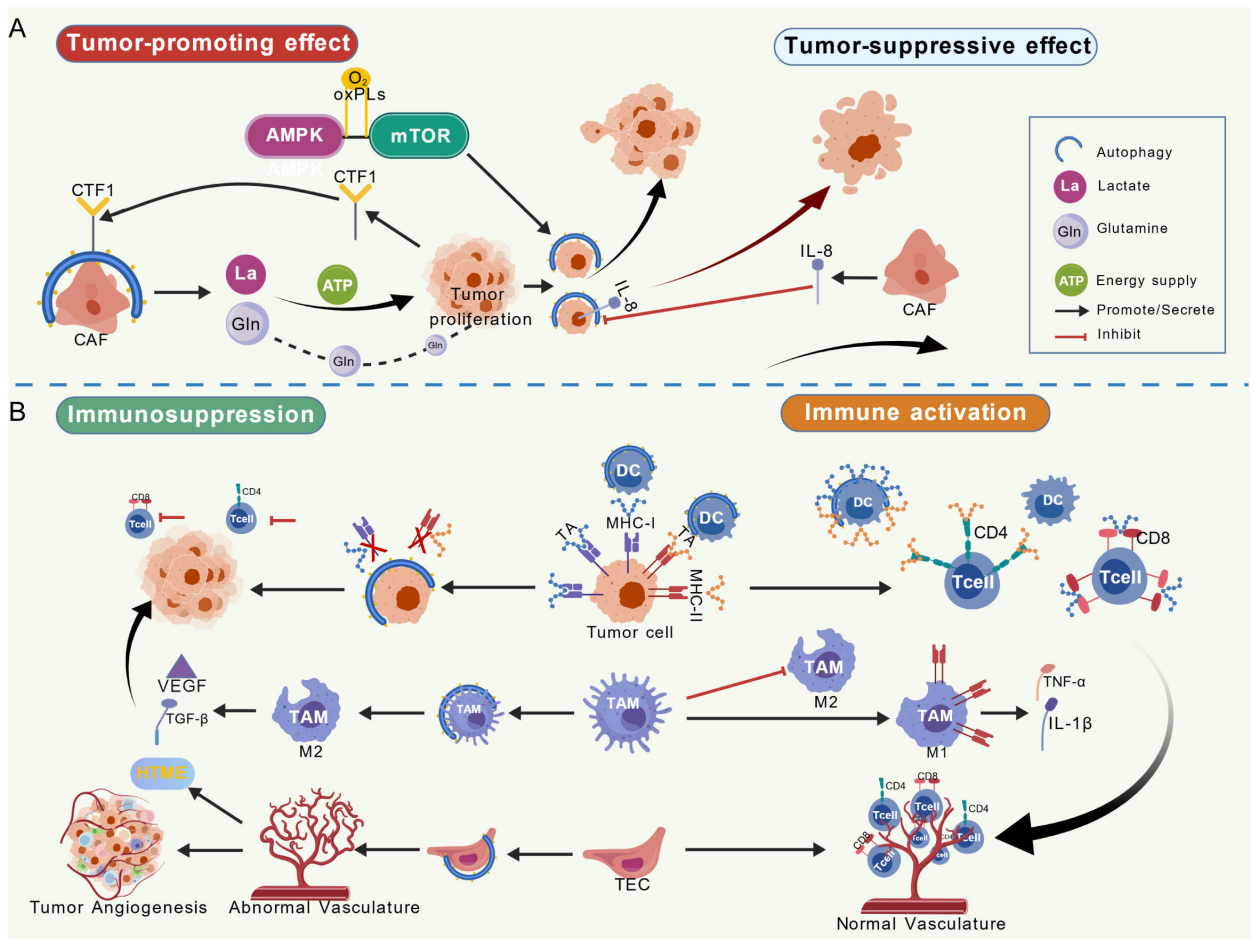
**(A)** Bidirectional regulation between tumor cells and stromal cells: Autophagy in stromal cells (e.g., CAFs) produces metabolites (lactate, glutamine) to support tumor proliferation; cytokines (IL-8, CTF1) mediate autophagy regulation between tumor cells and stromal cells. **(B)** Dual roles of autophagy in tumor-immune interactions: Autophagy promotes immune suppression by degrading MHC molecules, driving M2 polarization, and upregulating PD-L1; it enhances immune activation by improving DC antigen presentation, restoring NK cell function, and optimizing tumor vasculature to promote T cell infiltration. Created with BioGDP.com (158).

Autophagy mediates tumor-stromal cell crosstalk, controlling tumor progression via metabolic support and cytokine signal transduction (**Figure 7A**). Under stress conditions (nutrient deprivation, hypoxia), tumor cells and stromal cells regulate metabolism via autophagy to meet energy requirements. Autophagy in stromal cells (e.g., CAFs) produces metabolites (lactate, glutamine) that are taken up by tumor cells, providing a material basis for malignant proliferation (17, 19, 22). In the Drosophila intestinal tumor model, tumor cells can actively induce autophagy in adjacent non-neoplastic components, prompting them to release nutrients (e.g., amino acids) to meet their own metabolic needs (109). Oxidized phospholipids, as important metabolites in the TME, can induce autophagy through the AMPK-mTOR pathway, thereby promoting tumor EMT and metastatic potential (110). In addition, cytokine-mediated signaling pathways also regulate autophagy: in OC, CAF-secreted IL-8 inhibits cancer cell autophagy to promote migration (111); in BC, tumor-secreted cardiotrophin 1 (CTF1) activates fibroblast autophagy, further promoting fibrosis and tumor cell migration/metastasis (112).

#### 3.3.2 Autophagy in tumor-immune interactions

Autophagy plays dual roles in tumor-immune interactions, contributing to both immune suppression/escape and immune activation/antitumor responses (**Figure 7B**).

Immune suppression and escape: Tumor cells use cell-autonomous autophagy to degrade MHC class molecules, impairing antigen presentation and reducing immune recognition efficiency (17, 18, 27). Autophagy also drives TAMs polarization toward the immunosuppressive M2 phenotype. In an Lmdd-MPFG vaccine-treated HCC model, TAM M2 polarization depended on autophagy activation; autophagy inhibitors decreased M2 cells, increased antitumor M1 cells and effector CD8^+^ T cell infiltration, while the autophagy activator rapamycin promoted M0/M2-to-M1 conversion (113). Autophagy regulates tumor PD-L1 expression in a context-dependent manner: the Lmdd-MPFG vaccine upregulates PD-L1 in HCC cells via autophagy to enhance immunosuppression (113), while black phosphorus nanosheets (BPNS) induce autophagy in GBM to promote PD-L1 degradation and inhibit the PI3K-AKT pathway, reducing PD-L1 mRNA levels (114); chloroquine (CQ) or the NF-κB inhibitor ML120b blocks autophagy-mediated PD-L1 upregulation (113, 114). Moreover, tumor endothelial cells (TECs) activate autophagy under hypoxia to support survival; TECs in dysfunctional tumor vasculature show markedly increased autophagy, which maintains abnormal vessel structure, worsens hypoxia, limits immune cell infiltration, and promotes the STIME (105).

Immune activation and antitumor immunity: Autophagy remodels the tumor microenvironment toward an activation-prone state. Autophagy enhances dendritic cells (DCs) processing of tumor antigens and neoantigen exposure and, through MHC-I/II-mediated presentation, effectively initiates T cell responses (27). In PCa models, tumors suppress natural killer (NK) cell autophagy via the CXCL12-CXCR4-C/EBPβ axis, leading to mitochondrial dysfunction and reduced secretion of effector molecules (granzyme B, perforin); metformin-induced autophagy or Beclin1 overexpression restores NK cell cytotoxicity, and autophagy activation in CAR-NK cells further potentiates this effect (115). In GBM, BPNS can indirectly enhance NK cell recognition and killing efficiency (114). Inhibition of endothelial cell autophagy selectively eliminates dysfunctional tumor vasculature, optimizes vascular architecture and perfusion, promotes CD4^+^ and CD8^+^ T cell infiltration and perivascular immune-niche formation, and improves delivery of 5-Fluorouracil and anti-PD-1 antibodies; this effect is linked to p53 pathway activation and apoptosis of dysfunctional TECs (105).

## 4 Reciprocal regulation of NRP1 and autophagy in the TME

### 4.1 Correlation between NRP1 and ATGs expression and prognostic impact

In the TME, NRP1 plays a major transmembrane role. Autophagy, as a crucial intracellular degradation and recycling function, is closely related to NRP1. Pearson correlation analysis was performed to assess the expression association between NRP1 and key ATGs (correlation threshold |*r*| ≥ 0.5 and p-value < 0.05, a preliminary selection criterion referenced from canonical pan-cancer gene correlation studies in tumor biology). The result offers important clues for understanding the molecular mechanisms by which NRP1 may regulate autophagy. However, correlation does not imply causation or direct regulatory interaction. Subsequent functional validation using cell-based assays and animal models is required to clarify the mechanistic interplay between the two. NRP1 is overexpressed in many solid tumors. It not only participates in tumor growth and metastasis but also is involved in autophagy regulation, mediating the sensitivity and resistance to antitumor drugs, which is closely correlated with poor patient prognosis.

**Figure 8:**
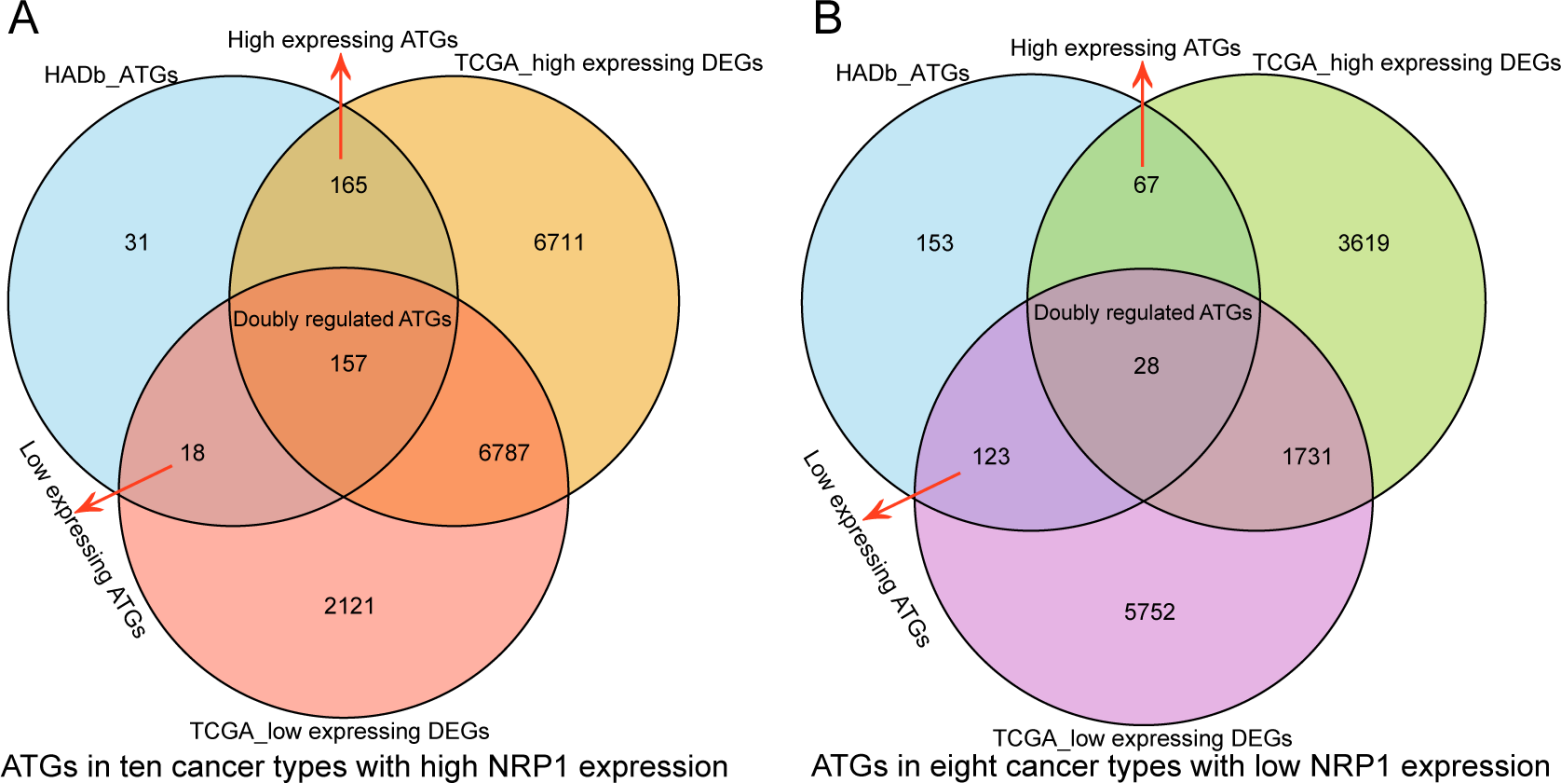
**(A, B)** Venn diagrams of DEGs and ATGs. **(A)** For 10 cancer types with NRP1 upregulation, 340 differentially expressed ATGs were identified (165 upregulated, 18 downregulated, 157 doubly regulated); **(B)** For 8 cancer types with NRP1 downregulation, 218 differentially expressed ATGs were found (67 upregulated, 123 downregulated, 28 doubly regulated).

**Figure 9:**
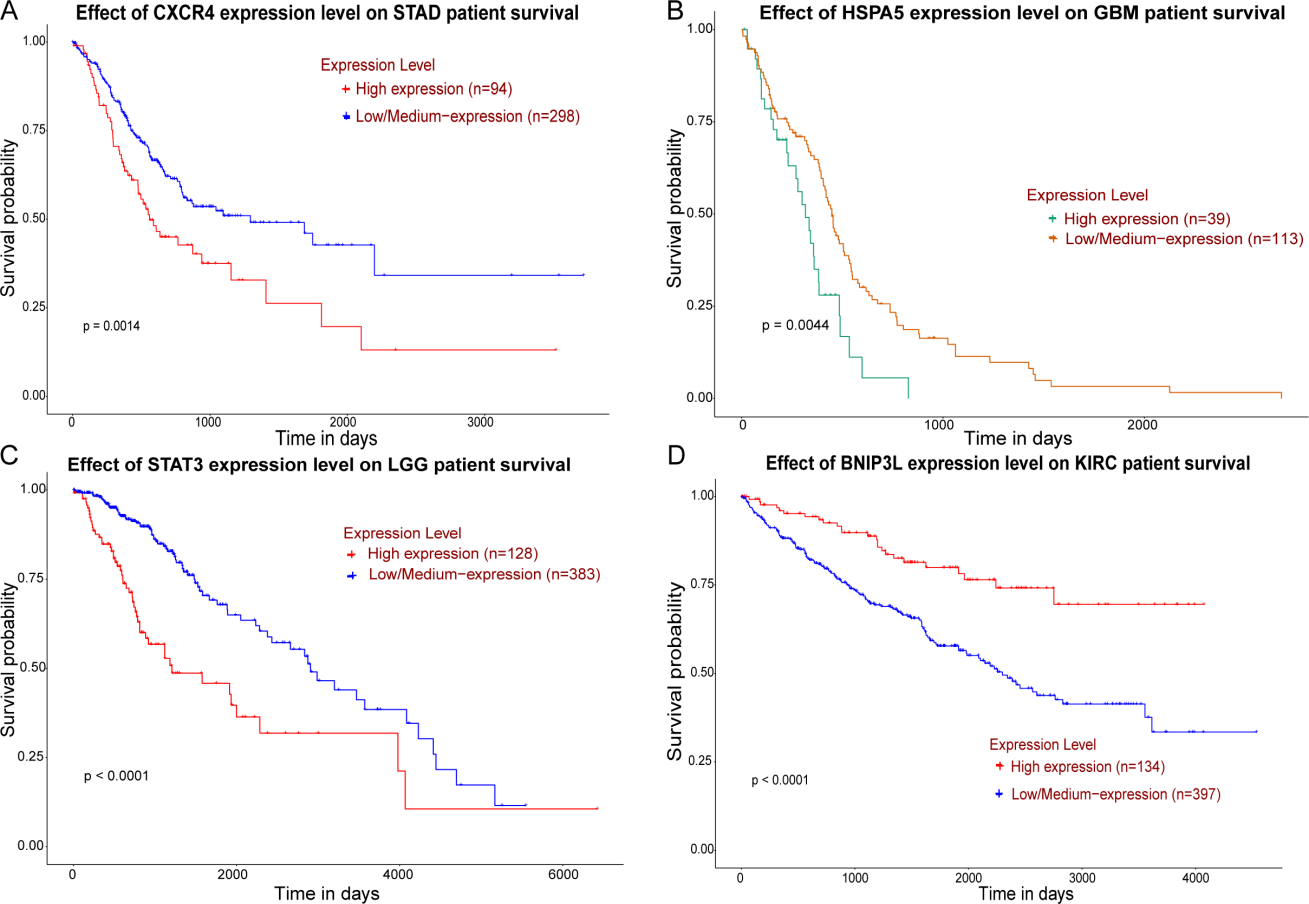
**(A–D)** Kaplan–Meier survival analysis curves of key ATGs from UALCAN platform: **(A)** CXCR4 high expression correlates with poor STAD prognosis; **(B)** HSPA5 high expression correlates with poor GBM prognosis; **(C)** STAT3 high expression correlate with poor LGG prognosis; **(D)** BNIP3L high expression correlate with favorable KIRC prognosis; Survival analysis curves for the remaining key ATGs are provided in the **Supplementary Materials**; A p-value < 0.05 is considered statistically significant.

We extracted 371 ATGs from the HADb database and retrieved differentially expressed genes (DEGs) associated with NRP1 across 18 cancer types from the GEPIA3 database, defining significant upregulation as log2FC ≥ 1 and q-value < 0.05, and significant downregulation as log2FC ≤ -1 and q-value < 0.05. All DEGs were identified using FDR-adjusted q values, with the correction threshold set at q < 0.05 to control for false positives arising from multiple testing. Using an R package to generate Venn diagrams, we identified the intersection between DEGs and ATGs to select key ATGs co-expressed with NRP1. The results showed that among the 10 cancer types with NRP1 upregulation, 340 differentially expressed ATGs were identified (165 upregulated, 18 downregulated, 157 bidirectionally expressed); among the 8 cancer types with NRP1 downregulation, 218 differentially expressed ATGs were identified (67 upregulated, 123 downregulated, 28 bidirectionally expressed) (**Figures 8A, B**). This provides clinical data supporting an association between NRP1 and the regulation of tumor cell autophagy within the TME. To examine the relationship between NRP1 and key ATGs and their links to survival outcomes, we performed Pearson correlation analyses for NRP1 and candidate ATGs using the GEPIA3 platform, selecting ATGs with |*r*| ≥ 0.5 and p < 0.05. Following standard TCGA-based quality-control criteria, we excluded genes with median TPM < 0.5 to enhance the reliability of the correlations. We then carried out Kaplan–Meier survival analyses on the UALCAN platform, using p < 0.05 as the threshold for significance, and identified the ATGs whose prognostic relevance associates with NRP1. The correlation analysis revealed that, in GC, GBM, LGG, and ccRCC, most ATGs show moderate to strong positive co-expression with NRP1, although their prognostic effects vary by cancer type. Specifically, the co-expression of CXCR4 and SEC23A with NRP1 in GC, HSPA5, P4HB, RAB13, and RELB with NRP1 in GBM, as well as STAT3, EIF2AK3, HSPA5, and TM9SF1 with NRP1 in LGG, was associated with a worse prognosis (p < 0.05) (**Figures 9A–C**). Conversely, in ccRCC, ATGs such as BNIP3, BNIP3L, LRRK2 and KDR, which co-expressed with NRP1, correlated with improved prognosis (p < 0.05) (**Figure 9D**). However, the above results represent only preliminary screening findings and require further experimental validation in future studies.

Bioinformatics analyses demonstrate that NRP1 is co-expressed with ATGs, and this co-expression correlates with clinical prognosis in solid tumors. In GC, CXCR4 showed a positive correlation with NRP1 (*r* = 0.527, p < 0.001), and high co-expression was associated with poorer overall survival (p = 0.0014). In GBM, HSPA5 correlated with NRP1 (*r* = 0.687, p < 0.001) and predicted worse prognosis (p = 0.0044). Detailed correlation coefficients and p-values for all analyzed ATGs are provided in **Table 3**. These co-expressed ATGs have been implicated in various tumor-related processes, yet their specific interplay with NRP1 in autophagy regulation remains largely unexplored. For instance, in GC, NRP1 and chemokine receptor 4 (CXCR4) are upregulated in tumor tissue compared to adjacent normal tissue, and both genes exhibit positive correlations with the cuproptosis-related gene FDX1, suggesting a potential association between immune infiltration and cuproptosis (116). Elevated expression of NRP1 and CXCR4 independently predicts poorer OS (HR = 2.025 and HR = 1.648, respectively) and is associated with advanced clinical stage, poor differentiation, and immune-related processes such as leukocyte migration and activation of the innate immune response (117). Previous studies have demonstrated that in GC, CXCR4 promotes tumor progression through multiple mechanisms, including the induction of immune suppression, maintenance of cancer stem cell characteristics, activation of downstream signaling pathways, and facilitation of metastasis. Its high expression can serve as a molecular marker for poor prognosis in GC patients. Targeting CXCR4 (e.g., the partial agonist TFF2-MSA) can provide new directions for GC treatment by reshaping the immune microenvironment, inhibiting CSC characteristics, and myelopoiesis, especially when used in combination with ICIs (118–120). However, there is currently no relevant research on the specific interaction mechanism between NRP1 and CXCR4 in GC, and whether it is related to the regulation of the autophagy process. Similarly, SEC23A in GC can promote tumor proliferation and progression, as well as reduce the efficacy of chemotherapy, by regulating the cell cycle, immune microenvironment, and endoplasmic reticulum stress-autophagy axis. Its high expression can serve as a potential biomarker and therapeutic target for poor prognosis in GC (121–123). No studies have reported an association between SEC23A expression and NRP1 in GC. In other cancer types, several ATGs co-expressed with NRP1 also lack documented interactions: in GBM, HSPA5, P4HB, RAB13, RELB; in LGG, STAT3, EIF2AK3, HSPA5, TM9SF1; and in ccRCC, BNIP3, BNIP3L, LRRK2 and KDR. The associations of these ATGs with NRP1 in the corresponding tumors lack research reports, and thus may be worthy directions for in-depth exploration in the future. Previous studies have established autophagy-related gene signatures with prognostic significance in multiple gastrointestinal cancers, including STAD, PAAD, COAD, and ESCA (124). The co-expression of NRP1 with key ATGs (e.g., CXCR4 in STAD, SEC23A in STAD) in these cancers suggests a potential link between NRP1 and autophagy regulation that warrants further investigation. Notably, many of the ATGs identified in our analysis overlap with those previously reported in autophagy-related prognostic models, reinforcing the hypothesis that NRP1 may participate in autophagy modulation across digestive tract malignancies.

**Table 3:** ATGs significantly correlated with NRP1 prognosis in cancer samples.

Cancer	ATG co-expression with prognosis	Pearson correlation analysis		Prognosis		Refs
		Correlation coefficient (*r*)	P-value	Result	P-value	
GC	CXCR4	0.527	6.57 × 10^−^³¹	Poor prognosis	0.0014	(116, 117)
	SEC23A	0.603	2.27 × 10^−4^²		0.0034	(121–123)
GBM	HSPA5	0.687	1.69 × 10^−24^	Poor prognosis	0.0044	NA
	P4HB	0.73	6.12 × 10^−29^		0.026	
	RAB13	0.516	1.16 × 10^−^¹²		0.028	
	RELB	0.501	6.03 × 10^−^¹²		0.017	
LGG	STAT3	0.593	4.39×10^−51^	Poor prognosis	< 0.0001	NA
	EIF2AK3	0.528	6.92×10^−39^		0.00016	
	HSPA5	0.535	3.81×10^−40^		0.0034	
	TM9SF1	0.543	2.01×10^−41^		< 0.0001	
ccRCC	BNIP3	0.594	5.5×10^−52^	Good prognosis	0.00032	NA
	BNIP3L	0.754	1.99 × 10^−98^		< 0.0001	
	KDR	0.812	9.43×10^−126^		0.00062	
	LRRK2	0.56	4.23×10^−45^		0.00042	

The default setting for Correlation Analysis on the GEPIA3 platform is to use the log_2_(TPM + 1) scale; Genes with extremely low expression (Median TPM < 0.5) and Pearson correlation coefficient |*r*| < 0.5 are filtered out; Kaplan–Meier survival analysis was performed using the UALCAN platform, and a p-value < 0.05 was considered statistically significant; NA: not available.

### 4.2 Regulatory mechanism between NRP1 and autophagy

NRP1 and autophagy interact in the TME. NRP1 both controls and is broken down by autophagy. These links influence tumor growth, metabolism, and how well drug therapies work. Here, we classify and label the strength of evidence for the aforementioned regulatory mechanisms (**Table 4**): Only CRC, GC, HCC, and OC currently have Level A direct mechanistic evidence for NRP1-autophagy interactions; for most other cancer types, the proposed regulatory relationships remain at the level of indirect clues (Level B) or correlative inferences (Level C) requiring experimental validation.

**Table 4:** Evidence matrix of NRP1-autophagy axis in major cancer types.

Cancer	Proposed mechanism	Evidence level	Experimental system	Strength assessment	Refs
CRC	EZH2-NRP1 inhibits autophagy	A (direct functional)	*in vitro* (cell lines), *in vivo* (xenograft)	Strong	(31)
GC	NRP1 activates Wnt/β-catenin to inhibit autophagy	A (direct functional)	*in vitro*, *in vivo*	Strong	(92)
HCC	Autophagy degrades NRP1; NRP1 status affects drug response	A (direct functional)	*in vitro*	Moderate	(32)
OC	NRP1 silencing restores sensitivity via AKT/autophagy	A (direct functional)	*in vitro*	Moderate	(70)
ccRCC	NRP1 indirectly modulates autophagy via VEGF/mTOR	B/C (indirect/correlative)	*in vitro* (cell lines), *in vivo* (mouse model)	Preliminary	(130)

#### 4.2.1 Metabolic stress facilitates the autophagic degradation of NRP1

Hypoxia or nutrient deprivation within the TME induces specific degradation of NRP1 via the autophagy-lysosome pathway. Studies demonstrate that exposure to 0.1% O_2_ or glucose deprivation significantly reduces NRP1 levels on the surface of endothelial and cancer cells, a process that can be inhibited by lysosomal or autophagy inhibitors (125). In HCC, lenvatinib exerts anti-tumor effects by inducing autophagy-mediated NRP1 degradation. Accumulation of NRP1 resulting from autophagy inhibition is a primary contributor to drug resistance, whereas hypoxia-induced HIF-1α sustains NRP1 levels through a degradation-compensation mechanism, thereby diminishing drug efficacy (32).

#### 4.2.2 NRP1 as a receptor for autophagy-targeted therapies

Leveraging the high expression of NRP1 in gliomas and melanomas, researchers have developed liposomes modified with R6dGR/R8dGR peptides to deliver autophagy inhibitors such as hydroxychloroquine (HCQ) with precision. In gliomas, NRP1-mediated delivery reverses the protective autophagy induced by ZD6474 (126), while in melanomas, this approach synergistically inhibits tumor metastasis by blocking autophagic flux (127). The relationship between NRP1 and autophagy is protocol-dependent. For instance, treatment of gliomas with TMZ combined with caffeine activates autophagy without altering NRP1 expression (128).

#### 4.2.3 NRP1 regulates autophagy through multiple signaling pathways with cancer-specific heterogeneity

NRP1 regulates autophagy through multiple pathways, exhibiting tumor-specific heterogeneity. Experimental evidence indicates that this heterogeneity manifests as differences in both molecular mechanisms and functional outcomes across different cancer types.

In GC, NRP1 suppresses autophagy and promotes proliferation by activating the Wnt/β-catenin pathway (92). Specifically, NRP1 stabilizes β-catenin and promotes its nuclear translocation, where it represses transcription of autophagy-related genes like LC3B and Beclin1. Knockdown of NRP1 in GC cells reduces β-catenin expression, increases the LC3-II/LC3-I ratio and autophagic vesicle formation, and decreases Ki67 expression. In xenograft models, NRP1 silencing inhibits tumor growth by restoring autophagy and suppressing β-catenin-mediated proliferation (92). This regulatory mechanism appears unique to GC, as it has not been observed in other cancer types. In CRC, NRP1 mediates two context-dependent regulatory mechanisms. EZH2-NRP1 axis in chemoresistance: The epigenetic regulator EZH2 binds the NRP1 promoter to enhance its transcription. Upregulated NRP1 activates the PI3K/AKT/mTOR pathway, leading to ULK1 phosphorylation and blockade of autophagy initiation. This results in reduced LC3b-II expression and decreased sensitivity of CRC cells (HCT116, SW480) to irinotecan. Knockdown of either EZH2 or NRP1 restores autophagic flux and reverses chemoresistance (31). NRP1-P62-NRF2-AKR1B10-GAPDH axis in metabolic stress adaptation: Under glucose starvation, NRP1 promotes cytoprotective autophagy to support cell survival. NRP1 knockdown upregulates P62, stabilizing NRF2 and driving AKR1B10 transcription. AKR1B10 then inhibits GAPDH nuclear translocation (a key autophagy step) via NADPH-dependent reduction. Conversely, NRP1 overexpression suppresses this cascade, releasing GAPDH to activate autophagy (129). This pathway is specific to metabolic stress and inactive under nutrient-rich conditions. In OC, NRP1 plays a context-dependent role in autophagy during PARP inhibitor (Olaparib) treatment. In partially sensitive OC cells (e.g., UWB-BRCA), Olaparib upregulates NRP1, which binds VEGFR2 to form a complex that inhibits AKT phosphorylation. This relieves AKT-mediated suppression of Beclin1, leading to moderate autophagy activation that promotes Olaparib sensitivity by facilitating DNA damage clearance (70). Conversely, in Olaparib-resistant OC cells (e.g., SKOV3) with high basal NRP1 expression, NRP1 overexpression inhibits autophagy via the PI3K/AKT pathway, reinforcing resistance. Silencing NRP1 or overexpressing miR-200c (a NRP1-targeting miRNA) restores autophagic flux and Olaparib sensitivity in resistant cells (70). In ccRCC, NRP1 indirectly regulates autophagy via the VEGF-NRP1-mTOR pathway, with contributions from TME components. As a co-receptor for VEGF, NRP1 enhances VEGF/VEGFR2 signaling to activate mTOR, which then phosphorylates ULK1 and Beclin1 to suppress autophagy and promote tumor cell proliferation (130). NRP1 expressed on tumor-associated endothelial cells and M2 macrophages further amplifies VEGF signaling, reinforcing autophagy inhibition in tumor cells (130). However, direct functional validation (e.g., NRP1 knockdown in specific TME cell types) is lacking, and the correlation between NRP1 expression and autophagy markers (LC3B, p62) in clinical ccRCC samples remains to be confirmed.

### 4.3 Mechanistic controversies and challenges

There are controversies regarding the interaction mode between NRP1 and autophagy, including regulation direction, pathway heterogeneity, and drug-induced effects. In CRC and GC, NRP1 is regarded as a negative regulator of autophagy (31, 92); while in HCC, NRP1 serves as a degradation substrate of autophagy (32); in some OC models, there is a lack of direct association between the two (70). The molecular pathways through which NRP1 regulates autophagy (such as Wnt, PI3K/AKT, AKR1B10, etc.) are highly diverse among different cancer types, and its regulatory sensitivity is influenced by the basal expression level of cell lines and the signal compensation ability (129). Not all chemotherapy-induced autophagy is dependent on NRP1. The effects of lenvatinib and irinotecan are closely related to NRP1 (31, 32), while olaparib, paclitaxel, etc., may trigger autophagy through non-NRP1-dependent pathways (70, 127). These contradictions are not irreconcilable; rather, they reflect the multidimensional complexity of the regulatory network and can be analyzed at the following four levels.

Differences in experimental systems and methodologies are the primary causes of the discrepant conclusions. HCC studies used a lenvatinib-plus-hypoxia pharmacological intervention focusing on protein degradation pathways and found that autophagy mediates lysosomal degradation of NRP1, hence characterizing NRP1 as an “autophagy substrate” (32). CRC studies employed a glucose-deprivation metabolic stress model to examine upstream regulation of autophagy by NRP1 and demonstrated that NRP1 promotes autophagy via the p62–NRF2 axis, thereby labeling NRP1 as an “autophagy regulator” (31). OC studies used an olaparib-induced DNA damage model and found that upregulated NRP1 alleviates autophagy suppression by inhibiting AKT phosphorylation, placing its function between that of a “regulatory factor” and a “non−substrate” (70). The variation in functional assignment arising from different intervention approaches highlights the experimental condition dependence of the NRP1–autophagy relationship.

The spatial heterogeneity of the tumor microenvironment further shapes the functional bias of NRP1. In GC and CRC, persistent nutrient deprivation and hypoxia within the TME compel tumor cells to actively regulate autophagy via NRP1 to maintain survival; accordingly, NRP1 primarily functions as an “autophagy regulator” (31, 92). In HCC, the microenvironment is characterized by the coexistence of highly vascularized regions and hypoxic areas; tumor cells in hypoxic zones degrade NRP1 via autophagy to adapt to stress, whereas this phenomenon is absent in normoxic regions, resulting in NRP1 acting simultaneously as both a “regulator” and a “substrate” within the same tumor (32). In ccRCC, the microenvironment is rich in fibrotic stromal components, and NRP1 promotes stromal fibrosis to form a physical barrier, thereby indirectly modulating autophagy to enhance tumor metabolic adaptation; this mechanism differs markedly from that observed in other cancer types (130).

Subtype-specific and subcellular compartmentalization of NRP1 also contribute to its functional dichotomy. NRP1 exists as two splicing isoforms: a full-length form (containing the MAM domain) and a truncated form (lacking the MAM domain). The full-length isoform is predominantly localized at the plasma membrane and regulates autophagy-related pathways via ligand binding (as observed in gastric cancer), whereas the truncated isoform is cytosolic and more readily recognized and degraded by autophagosomes (as observed in endothelial cells of hepatocellular carcinoma). In addition, differences in cellular compartments play a critical role: in tumor cells, NRP1 predominantly functions as a regulatory factor, while in vascular endothelial cells, NRP1 is more prone to serve as an autophagic substrate; this distinction is particularly pronounced in angiogenic regions of clear cell renal cell carcinoma.

Based on the above analysis, we propose a “time-dependent functional switching” hypothesis to reconcile existing contradictions: during early tumorigenesis, NRP1 acts as an autophagy regulatory factor that reduces DNA damage by suppressing autophagy, thereby promoting tumor initiation; in advanced stages of the tumor, persistent metabolic stress and therapeutic pressure hijack autophagy into a pro-survival mechanism, at which point NRP1 is degraded as an autophagy substrate—on one hand releasing its bound pro-tumor ligands (such as VEGF), and on the other hand providing nutrients to tumor cells through its own degradation.

Future research should deepen mechanistic investigation across multiple levels. At the molecular level, it is necessary to identify the specific adaptor proteins that mediate autophagic recognition of NRP1 (e.g., LC3-interacting motifs) and the regulatory patterns of molecular chaperones, and to explore how conformational changes of NRP1 induced by different ligands (VEGF165, SEMA3A) differentially regulate autophagy pathways. At the cellular level, attention should be paid to cell–type–specific regulatory differences: in tumor cells, NRP1 may directly regulate autophagy, whereas in immune cells (such as regulatory T cells and tumor-associated macrophages) and stromal cells, NRP1 may indirectly affect autophagy via paracrine signals. Secondary analyses of single-cell transcriptomic databases such as TISCH (https://tisch.compbio.cn/home/) for cancer types, including gastric cancer, pancreatic cancer, and glioblastoma, could be used to localize expression patterns of NRP1 and autophagy-related genes across distinct cellular subpopulations, providing evidence to refine the “cell type–dependent regulation” hypothesis. At the level of clinical translation, it is essential to elucidate the commonalities and specificities of NRP1–autophagy axis–mediated regulation across different cancers and its cooperative mechanisms with immune checkpoints such as PD-1/PD-L1, thereby laying the groundwork for developing precise therapeutic strategies.

## 5 Progress and challenges in targeted regulation of NRP1 and autophagy in cancer therapy

### 5.1 Targeting NRP1 and combination therapeutic strategies

NRP1, as a multifunctional co-receptor, has become a highly promising target in anti-tumor therapy due to its unique biological properties. Currently, a diversified system of therapeutic strategies targeting NRP1 has been established, including monoclonal antibodies, targeting peptides, small molecule inhibitors, and gene interventions. Moreover, the combined application of such targeted therapies with autophagy regulators, ICIs, or chemotherapy drugs has demonstrated significant synergistic anti-tumor potential (**Table 5**).

**Table 5:** Research progress on NRP1 targeted therapy strategies.

Treatment type	Therapeutic agents	Mechanism	Conditions	Progress in clinical research	Refs
Monoclonal antibody	TB-403	Blocks PIGF-NRP1 interaction, inhibiting tumor growth and metastasis.	Recurrent/refractory MB	Phase I completed (NCT02748135); safe and specific.	(132)
	MNRP1685A	Binds NRP1 b1/b2 domain; blocks VEGF binding and VEGFR2-NRP1 complex formation.	Advanced solid tumors	Phase I completed (NCT00747734); safe, additive effect with bevacizumab.	(131)
Small-molecule inhibitor	Pitavastatin	Inhibits NRP1/ZFX axis, downregulates MDR-related proteins (P-gp, ABCC1), reversing multidrug resistance.	CRC(HCT116L)/NSCLC(A549/DDP)	Preclinical (2025); reverses oxaliplatin/cisplatin resistance, enhances chemotherapy efficacy.	(133)
	Cordycepin	Binds NRP1 b1/b2 domains; downregulates NRP1 expression.	Lung cancer cell line H1975	Preclinical (2023); strong clinical translation potential.	(134)
Targeted peptide	CEND-1	Binds αV integrins (RGD) and NRP1 (CendR); enhances chemotherapy delivery.	Advanced PDAC	Phase I completed (NCT03517176); improves drug delivery, safe.	(136)
	Fmoc-Gffy-AP-CK2	CK2 binds NRP1, enabling nanoparticle delivery; induces apoptosis via caspase3/GSDME; synergizes with PD-1 inhibitors.	Multiple human cancer cell lines	Preclinical (2025); inhibits tumor growth, enhances PD-1 therapy.	(137)
Gene intervention	MiRNA-19b-3p	Targets NRP1 3’-UTR, downregulating its expression.	GC	Preclinical (2020); inhibits proliferation/invasion, silencing NRP1 suppresses tumor growth.	(135)
	MiR-200c	Binds NRP1 3’-UTR, downregulating expression; enhances olaparib sensitivity in resistant ovarian cancer.	OC	Preclinical (2020); restores olaparib sensitivity in resistant cells.	(70)
	EZH2	Binds NRP1 promoter, enhancing transcription; inhibits autophagy via EZH2-NRP1 axis, driving irinotecan resistance.	CRC	Preclinical (2025); silencing EZH2 or NRP1 restores irinotecan sensitivity.	(31)

Clinical trials from: clinicaltrials.gov; MDR-related proteins: Multidrug resistance proteins.

Phased progress has been made in the research on monoclonal antibodies targeting NRP1. Among them, MNRP1685A can effectively inhibit tumor angiogenesis and reduce the phosphorylation level of AKT by blocking the VEGF-NRP1 signaling axis (33, 131); TB-403 shows good safety and disease-stabilizing effects in medulloblastoma patients by interfering with the placental growth factor (PlGF) and its receptor NRP1 (132). Small-molecule inhibitors of NRP1 have also emerged; Pitavastatin can reverse tumor drug resistance by directly binding to NRP1 (133), while Cordycepin can exert dual anti-tumor and immunomodulatory effects by downregulating NRP1 expression (134). Genetic and peptide tools also have important application values in NRP1-targeted therapy. miR-19b-3p and miR-200c can downregulate NRP1 expression through the RNA interference mechanism, thereby inhibiting EMT or enhancing olaparib-induced tumor cell apoptosis (70, 135). When the targeted peptide CEND-1 is combined with chemotherapy drugs, it shows encouraging efficacy in the treatment of advanced PDAC (136); the NRP1-targeted peptide Fmoc-Gffy-AP-CK2 can not only directly kill tumors by inducing tumor cell pyroptosis but also enhance the therapeutic response of PD-1 inhibitors (137).

The combined strategy of NRP1 targeted therapy and autophagy regulation is prominent in reversing tumor drug resistance. In CRC, EZH2 can drive NRP1 expression, thereby suppressing autophagy and enhancing irinotecan resistance; knocking down NRP1 can relieve its inhibitory effect on autophagy, increasing the killing effect of irinotecan on tumor cells by 20%-30% (31). Moreover, in the combination therapy strategy, targeted NRP1 therapy can exert a synergistic anti-tumor effect by remodeling the TME. When combined with ICIs, it can effectively relieve the immunosuppression mediated by Tregs and significantly enhance the infiltration level of IFN-γ^+^ CD8^+^ T cells in tumor tissues (74, 76). In a clinical trial of advanced melanoma resistant to ICIs, the treatment of a dendritic cell vaccine targeting NRP1 combined with Dasatinib showed that 46% (6/13) of the evaluable patients had good tolerance and produced a synergistic immunological/clinical response (138).

### 5.2 Targeting autophagy and combination therapeutic strategies

Autophagy exhibits a significant dual regulatory role in tumorigenesis and development, making it a highly promising key intervention target in the field of cancer treatment. Currently, therapeutic strategies targeting autophagy are mainly divided into two major categories: inhibiting the protective autophagy of tumor cells to enhance their sensitivity to treatment, or inducing cytotoxic autophagy in tumor cells to directly kill tumors, which shows remarkable synergistic potential, especially in combination therapies (**Table 6**).

**Table 6:** Progress in clinical research of autophagy modulators in cancer treatment.

Treatment type	Therapeutic agents or drugs	Conditions	Progress in clinical research	Refs
Autophagy inhibitor	CQ	DCIS	Phase I/II completed (NCT01023477); safe	(139)
	HCQ +Chemotherapy (Vorinostat)	Refractory mCRC	Phase II completed (NCT02316340); safe	(140)
	HCQ + Targeted therapy (Trametinib + Dabrafenib)	Advanced BRAF mutant Melanoma	Phase I/II completed (NCT02257424); safe	(142)
	CQ + ICI (Atezolizumab)	Osteosarcoma	Preclinical study (2021)	(143)
	HCQ + ICI (Atezolizumab) +Targeted therapy (Cobimetinib)	KRAS-mutated advanced malignancies	Phase I/II (NCT04214418); Terminated due to toxicity and lack of efficacy.	None
Autophagy inhibitor	mTOR inhibitor (Nab-Rapamycin)	Multiple solid tumors with mTOR activation mutations	Phase I/II completed (NCT03190174); safe	(147)
	mTOR inhibitor (Lomitapide) + Mouse PD-1 antibody (RMP1-14)	CRC	Preclinical study (2022)	(152)
	ABTL0812	Advanced solid tumors	Phase I completed (NCT02201823); safe	(146, 151)
	Ibrilatazar (ABTL0812) + Chemotherapy (Paclitaxel + Carboplatin)	Advanced EC or sq-NSCLC	Phase I/II completed (NCT03366480); safe	(149, 150)
	LAM561 (2-OHOA)	Advanced solid tumors including glioma	Phase I/II completed (NCT01792310); safe	(148)
	Sunitinib + anti-CTLA-4 antibody	Melanoma/lung cancer	Preclinical study (2021)	(153)

Clinical trials from: clinicaltrials.gov; None: Due to the early termination of the trial, no relevant references are available.

The core objective of autophagy inhibition strategies is to overcome tumor resistance induced by chemotherapy and targeted therapy, and its clinical exploration has been carried out in various tumor types. In the aspect of combined chemotherapy, studies related to ductal carcinoma *in situ* (DCIS) have shown that chloroquine (CQ) can restore the expression level of caveolin-1 (Cav-1) in CAFs by inhibiting the autophagy/lysosome degradation pathway, thereby reversing the pro-tumor phenotype of CAFs (139). In metastatic colorectal cancer (mCRC) and small cell lung cancer (SCLC), HCQ combined with chemotherapy regimens has demonstrated good safety, but the survival benefits (PFS/OS) of patients still need to be further verified through the precise matching of subjects by biomarker screening (140, 141). In the field of combined targeted drugs, after BRAF-mutant melanoma patients received HCQ combined with dabrafenib/trametinib, the objective response rate (ORR) reached 85%, and the prognosis of patients was significantly improved (142). In addition, autophagy inhibition can also promote the infiltration of T cells into the tumor microenvironment by upregulating chemokines such as CXCL10, thereby enhancing the efficacy of anti-PD-1/PD-L1 immunotherapy (143–145).

Autophagy induction strategies achieve direct killing of drug-resistant tumor cells by triggering cytotoxic autophagy in tumor cells. Meanwhile, they can enhance the therapeutic effect by reshaping the tumor immune microenvironment. In terms of single-drug exploration, ABTL0812 (which acts by blocking the AKT-mTOR axis) and 2-hydroxyoleic acid (2-OHOA) have shown good tolerance and long-term disease stabilization ability in solid tumors and gliomas (146–148). In the combined application with chemotherapy, after patients with endometrial cancer and squamous cell lung cancer received ABTL0812 combined with carboplatin/paclitaxel treatment, the ORR reached 32.5% and 65.8% respectively (149, 150). Moreover, in preclinical models of TNBC, ABTL0812 can also enhance the therapeutic efficacy of paclitaxel and reverse its chemotherapy resistance (151). The combined application of autophagy inducers and ICIs also shows broad prospects. When the mTORC1 inhibitor lomitapide is used in combination with the anti-PD-1/PD-L1 antibody (RMP1-14) in animal models of MC38 colorectal cancer and B16-F10 melanoma, it exhibits a significant synergistic anti-tumor effect (152). In addition, drugs such as sunitinib can enhance the body’s anti-tumor immune activity by inducing the autophagic degradation of PD-L1 (153).

### 5.3 Challenges in clinical translation

At present, research directly targeting the NRP1-autophagy axis remains in its infancy, with limited studies available. The existing evidence is derived solely from preclinical investigations, with a conspicuous absence of clinical translation data. To translate the NRP1-autophagy axis targeted therapy from the laboratory to the clinic, multiple challenges such as drug development, efficacy evaluation, patient selection, and drug resistance need to be overcome.

Most of the existing NRP1-targeted drugs target the b1/b2 domain, which may simultaneously block the signals of multiple ligands such as VEGF and PlGF, leading to off-target effects (131, 154); the autophagy inhibitor HCQ is prone to cause hematological toxicity in combination therapy (141). Developing highly selective, low-toxicity, and high-efficiency targeted drugs remains an urgent problem to be solved. Most of the existing biomarker models, such as NRP1+ATGs and p62+LAMP1, are largely based on small sample sizes or retrospective studies, lacking validation from large-scale prospective clinical trials (153, 155); the cutoff values of NRP1 vary across different cancer types, making it difficult to directly apply them to clinical patient stratification (57, 156). Moreover, tumor heterogeneity results in substantial interpatient variability in therapeutic responses. Critically, the relationship between NRP1 and autophagy is not invariant: in CRC and GC, NRP1 functions as a “negative regulator of autophagy” and thereby promotes tumor progression (31, 92); conversely, in HCC, NRP1 serves as an “autophagic degradation substrate,” and its degradation has been implicated in mediating resistance to lenvatinib (32); in ovarian cancer, its role varies depending on the pharmacological context (70). This functional plasticity implies that therapeutic strategies validated in one tumor type may fail or even produce adverse effects when directly applied to another cancer type or to distinct intratumoral regions of the same tumor (e.g., hypoxic versus normoxic zones in HCC). For example, in HCC, tumor cells in hypoxic regions degrade NRP1 via autophagy, whereas cells in normoxic regions exhibit high NRP1 expression (32); consequently, NRP1 expression and autophagic activity may differ among tumor subtypes within the same patient, complicating therapeutic intervention. It is necessary to combine single-cell RNA sequencing and spatial omics technologies to analyze the heterogeneity of the NRP1-autophagy axis and formulate dynamic treatment strategies (11, 102).

Combination therapy regimens need to balance synergistic effects and toxicity control: in CRC, the combination of an EZH2 inhibitor and an NRP1 inhibitor can downregulate NRP1 expression from the upstream and enhance the effect of autophagy activation (31); in PDAC, the combination of the NRP1-targeted peptide CEND-1 and chemotherapy can improve drug delivery and reduce the matrix barrier, but further clinical trials are needed to optimize the dosage and administration sequence (136). Most of the existing NRP1-targeted therapy studies are in phase I/II clinical trials, with small sample sizes and a lack of unified efficacy evaluation indicators; the functions of autophagy vary significantly across different tumor types and their development stages, making it urgent to develop individualized treatment strategies (142, 157).

An additional challenge lies in choosing the optimal modality to target NRP1. Monoclonal antibodies and ligand-based blockers (e.g., peptides) can effectively inhibit extracellular ligand binding but may not fully abrogate intracellular signaling cascades. In contrast, genetic approaches (siRNA, miRNA) or small molecules targeting NRP1 expression could achieve more comprehensive inhibition, yet face hurdles in specific delivery and off-target effects. Given that NRP1 is a transmembrane glycoprotein with endocytic properties, it can be exploited for targeted drug delivery: for instance, the CEND-1 peptide utilizes NRP1-mediated internalization to enhance chemotherapy penetration in tumors (136). Future strategies should combine NRP1-targeted delivery with cargoes that modulate autophagy or other pathways, and optimize nanoparticle formulations (e.g., liposomes, polymeric nanoparticles) to improve biodistribution and reduce toxicity. Additionally, leveraging the CendR motif to enhance tumor penetration and using dual-targeting approaches may further refine therapeutic index.

## 6 Conclusions and outlook on future directions

The NRP1-autophagy axis functions as a critical hub within the TME that regulates tumor progression and treatment response, offering a promising therapeutic target for precision oncology. Although challenges remain—most notably cancer-type specificity, the dual roles of autophagy, and barriers to clinical translation—future research that concentrates on two core directions and purposefully addresses these bottlenecks could facilitate the clinical translation of NRP1-autophagy-targeted therapies and achieve transformative advances in the field.

At the mechanistic level, integrate cutting-edge technologies such as single-cell sequencing and spatial omics to systematically map the expression profiles and spatial distributions of NRP1 and key ATGs across tumor cells, Tregs, TAMs, CAFs, and endothelial cells in clinically poor-prognosis malignancies such as GC, GBM, and PDAC, thereby elucidating their cell-type-specific functions and spatiotemporal regulatory patterns. At the therapeutic level, prioritize the development of mechanism-based dual-targeted and combination intervention strategies and investigate their synergistic effects with immunotherapy: at the drug development level, design bispecific molecules capable of simultaneously interfering with NRP1 extracellular ligand binding and intracellular autophagy-regulatory signaling to achieve precise and efficient targeting of the NRP1-autophagy axis; at the combination regimen level, validate mechanism-guided combination therapies and further optimize dosing and scheduling; at the immune-synergy level, perform in-depth analyses of the cooperative mechanisms between the NRP1-autophagy axis and immune checkpoint inhibitors to define the optimal combination modalities, doses, and applicable cancer types, thereby providing new strategies to overcome tumor resistance to immunotherapy.
