# Supplementary material for: Crosstalk between NRP1 and autophagy in the tumor microenvironment: from molecular mechanisms to therapeutic targeting
Source: Front Immunol. 2026 Mar 23;17:1786502. doi: 10.3389/fimmu.2026.1786502 (PMC13050948; doi:10.3389/fimmu.2026.1786502)
Supplement: Supplementary file 1 [file DataSheet1.docx]

Supplementary Material

# Supplementary Data

R code for generating a Venn diagram：

install.packages("VennDiagram")

library(dplyr)

library(VennDiagram)

dir.deg <- "D:/"

hadb.file <- file.path(dir.deg, "HADb.csv")

dir.out <- dir.deg

hadb <- read.csv(hadb.file, stringsAsFactors = FALSE)

autophagy.genes <- unique(hadb$name)

cancers <- c("BRCA","CESC","CHOL","DLBC","ESCA","GBM","KICH","KIRC",

"LAML","LGG","LUSC","OV","PAAD","READ","SARC","STAD","UCEC","UCS")

high.group <- c("CHOL","DLBC","STAD","ESCA","GBM","KIRC","LAML","LGG","PAAD","SARC")

low.group <- setdiff(cancers, high.group)

for (can in cancers) {

deg.file <- file.path(dir.deg, paste0(can, "_DEGs.csv"))

if (!file.exists(deg.file)) next

deg <- read.csv(deg.file, stringsAsFactors = FALSE)

over <- deg %>% filter(log2FC > 1, q.value <= 0.05)

low <- deg %>% filter(log2FC < -1, q.value <= 0.05)

write.csv(over, file.path(dir.out, paste0("overexpressing_", can, "_DEGs.csv")), row.names = FALSE)

write.csv(low, file.path(dir.out, paste0("lowexpressing_", can, "_DEGs.csv")), row.names = FALSE)

arg.over <- over %>% filter(name %in% autophagy.genes)

arg.low <- low %>% filter(name %in% autophagy.genes)

write.csv(arg.over, file.path(dir.out, paste0("ATGs_overexpressing_", can, "_DEGs.csv")), row.names = FALSE)

write.csv(arg.low, file.path(dir.out, paste0("ATGs_lowexpressing_", can, "_DEGs.csv")), row.names = FALSE)

}

high.over.list <- lapply(high.group, function(can){

f <- file.path(dir.out, paste0("overexpressing_", can, "_DEGs.csv"))

if (file.exists(f)) read.csv(f) else data.frame()

})

high.low.list <- lapply(high.group, function(can){

f <- file.path(dir.out, paste0("lowexpressing_", can, "_DEGs.csv"))

if (file.exists(f)) read.csv(f) else data.frame()

})

NRP1_high_over <- bind_rows(high.over.list) %>% distinct(name, .keep_all = TRUE)

NRP1_high_low <- bind_rows(high.low.list) %>% distinct(name, .keep_all = TRUE)

write.csv(NRP1_high_over, file.path(dir.out, "NRP1_high_overexpressing_DEGs.csv"), row.names = FALSE)

write.csv(NRP1_high_low, file.path(dir.out, "NRP1_high_lowexpressing_DEGs.csv"), row.names = FALSE)

low.over.list <- lapply(low.group, function(can){

f <- file.path(dir.out, paste0("overexpressing_", can, "_DEGs.csv"))

if (file.exists(f)) read.csv(f) else data.frame()

})

low.low.list <- lapply(low.group, function(can){

f <- file.path(dir.out, paste0("lowexpressing_", can, "_DEGs.csv"))

if (file.exists(f)) read.csv(f) else data.frame()

})

NRP1_low_over <- bind_rows(low.over.list) %>% distinct(name, .keep_all = TRUE)

NRP1_low_low <- bind_rows(low.low.list) %>% distinct(name, .keep_all = TRUE)

write.csv(NRP1_low_over, file.path(dir.out, "NRP1_low_overexpressing_DEGs.csv"), row.names = FALSE)

write.csv(NRP1_low_low, file.path(dir.out, "NRP1_low_lowexpressing_DEGs.csv"), row.names = FALSE)

high.over.name <- NRP1_high_over$name

high.low.name <- NRP1_high_low$name

venn.plot.high <- draw.triple.venn(

area1 = length(autophagy.genes),

area2 = length(high.over.name),

area3 = length(high.low.name),

n12 = sum(autophagy.genes %in% high.over.name),

n23 = sum(high.over.name %in% high.low.name),

n13 = sum(autophagy.genes %in% high.low.name),

n123 = sum(autophagy.genes %in% intersect(high.over.name, high.low.name)),

category = c("HADb", "NRP1_high_over", "NRP1_high_low"),

fill = c("skyblue", "orange2", "tomato"),

cex = 1.2, cat.cex = 1.2

)

pdf(file.path(dir.out, "Venn_high_groups.pdf"), width=7, height=7)

grid.draw(venn.plot.high)

dev.off()

low.over.name <- NRP1_low_over$name

low.low.name <- NRP1_low_low$name

venn.plot.low <- draw.triple.venn(

area1 = length(autophagy.genes),

area2 = length(low.over.name),

area3 = length(low.low.name),

n12 = sum(autophagy.genes %in% low.over.name),

n23 = sum(low.over.name %in% low.low.name),

n13 = sum(autophagy.genes %in% low.low.name),

n123 = sum(autophagy.genes %in% intersect(low.over.name, low.low.name)),

category = c("HADb", "NRP1_low_over", "NRP1_low_low"),

fill = c("skyblue", "olivedrab3", "orchid3"),

cex = 1.2, cat.cex = 1.2

)

pdf(file.path(dir.out, "Venn_low_groups.pdf"), width=7, height=7)

grid.draw(venn.plot.low)

dev.off()

message("=== 全部完成！请检查输出文件夹中的 CSV 与 pdf 文件 ===")

make_ARGs_set <- function(prefix, dir.out, hadb_genes) {

over_df <- read.csv(file.path(dir.out, paste0(prefix, "_overexpressing_DEGs.csv")))

low_df <- read.csv(file.path(dir.out, paste0(prefix, "_lowexpressing_DEGs.csv")))

arg_over <- over_df %>% filter(name %in% hadb_genes)

arg_low <- low_df %>% filter(name %in% hadb_genes)

double <- arg_over %>%

inner_join(arg_low, by = "name", suffix = c("_over", "_low")) %>%

select(name) %>%

inner_join(arg_over, by = "name")

only_over <- anti_join(arg_over, double, by = "name")

only_low <- anti_join(arg_low, double, by = "name")

write.csv(arg_over,

file.path(dir.out, paste0(prefix, "_ATGs_overexpressing_DEGs.csv")),

row.names = FALSE)

write.csv(arg_low,

file.path(dir.out, paste0(prefix, "_ATGs_lowexpressing_DEGs.csv")),

row.names = FALSE)

write.csv(double,

file.path(dir.out, paste0(prefix, "_double_ATGs_DEGs.csv")),

row.names = FALSE)

write.csv(only_over,

file.path(dir.out, paste0(prefix, "_ATGs_only_overexpressing_DEGs.csv")),

row.names = FALSE)

write.csv(only_low,

file.path(dir.out, paste0(prefix, "_ATGs_only_lowexpressing_DEGs.csv")),

row.names = FALSE)

message(paste(prefix, "组 5 个 ARGs 文件已生成"))

}

make_ARGs_set("NRP1_high", dir.out, autophagy.genes)

make_ARGs_set("NRP1_low", dir.out, autophagy.genes)

message("=== 全部 12 个 ARGs 相关文件已输出到：", dir.out, " ===")

# Supplementary Figures and Tables

For more information on Supplementary Material and for details on the different file types accepted, please see [here](https://www.frontiersin.org/guidelines/author-guidelines#supplementary-material).

## Supplementary Figures


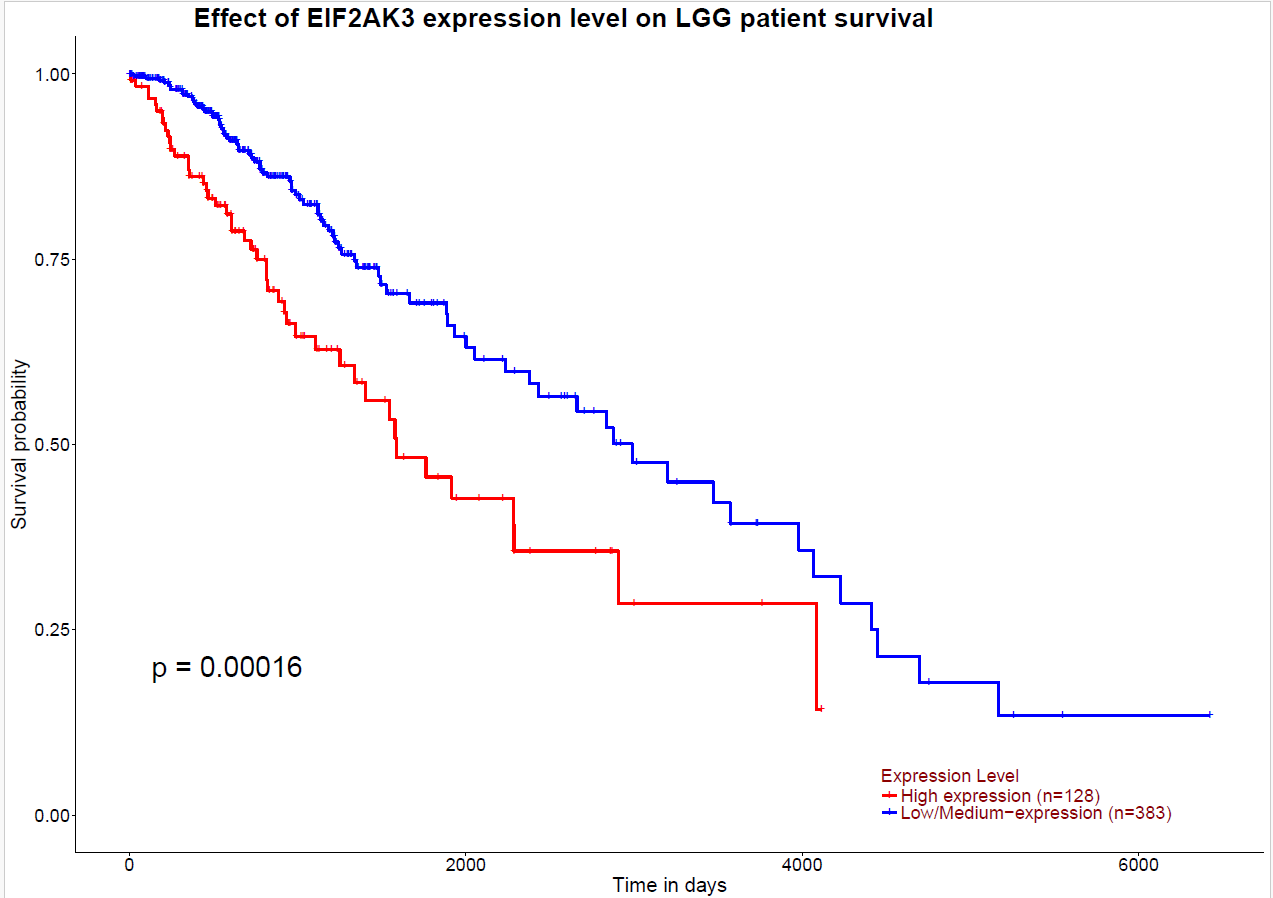

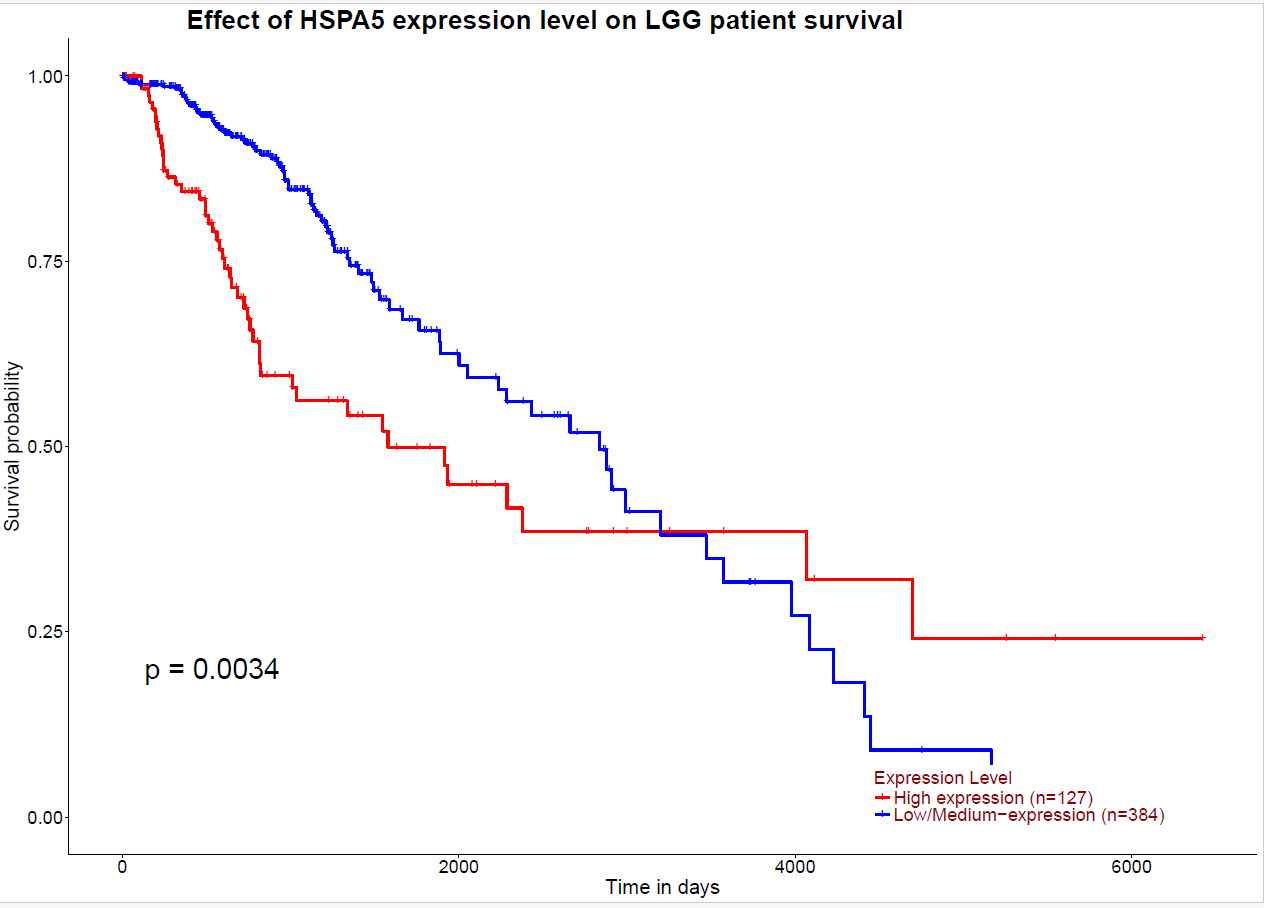


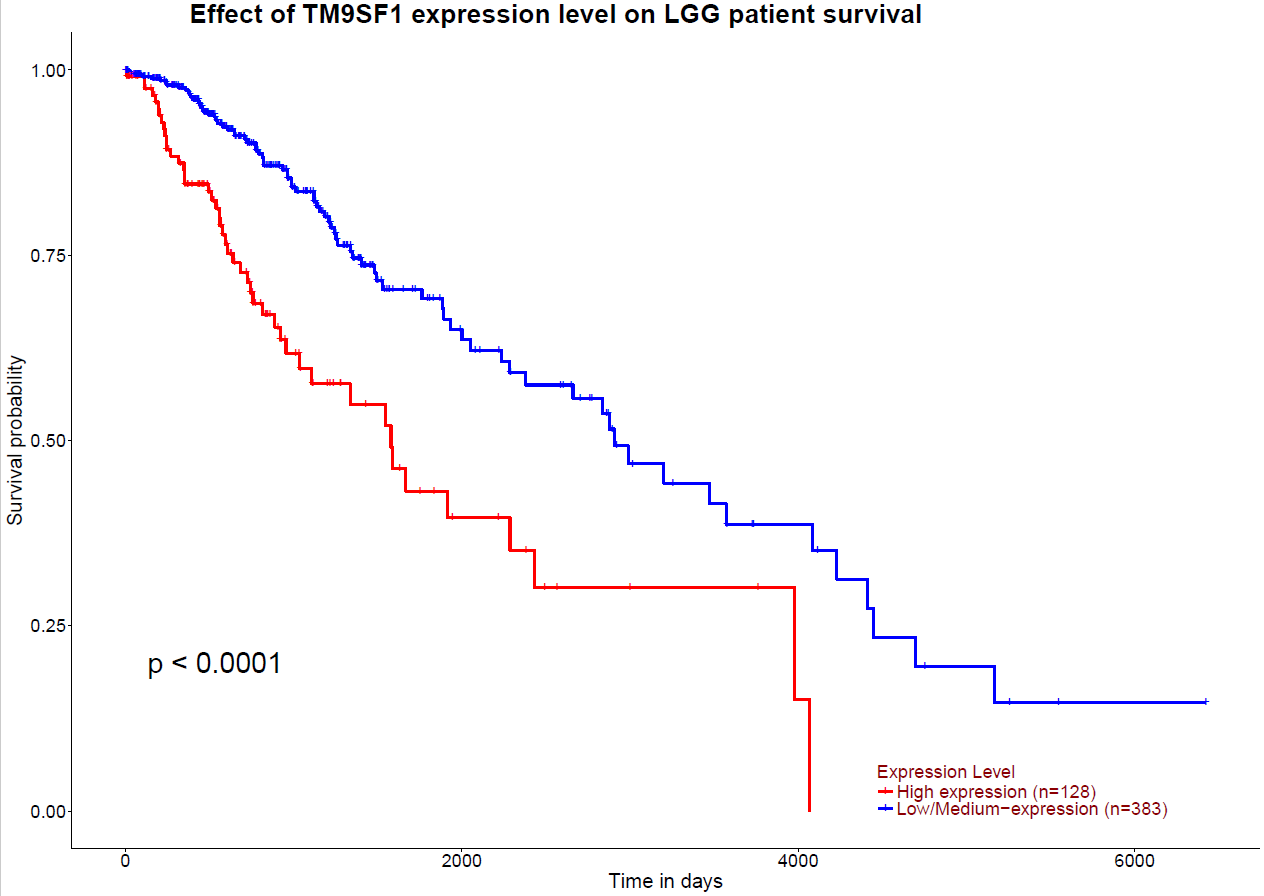

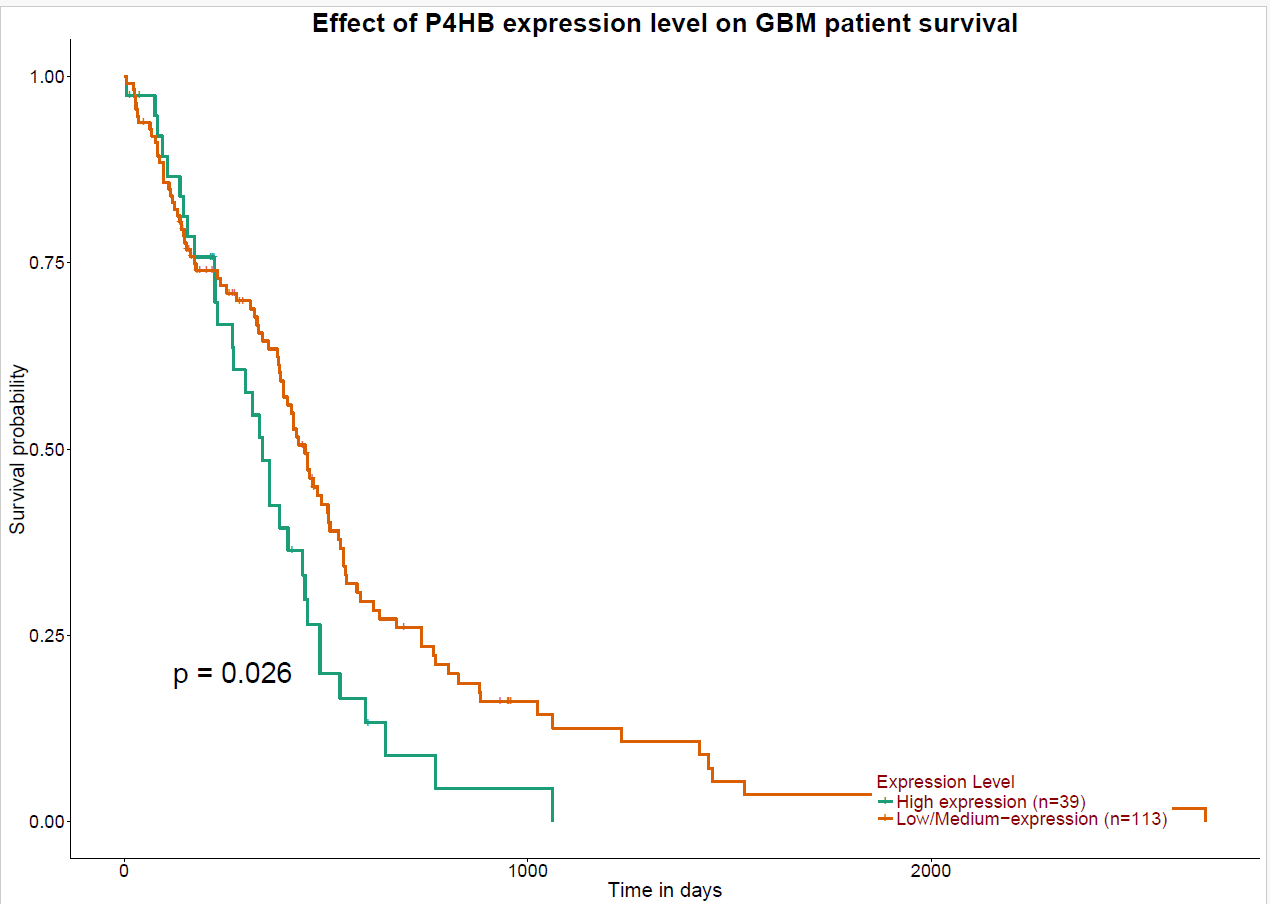


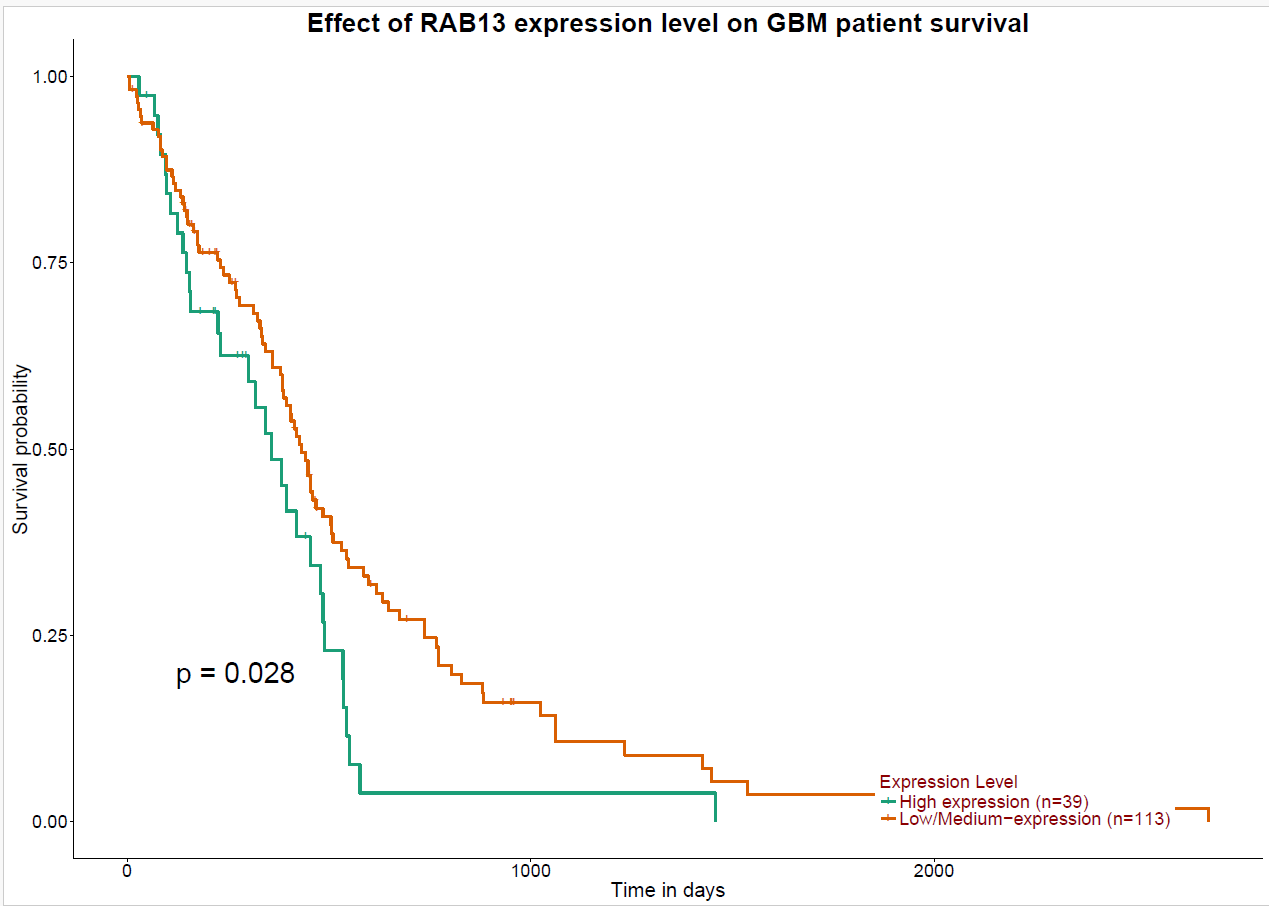

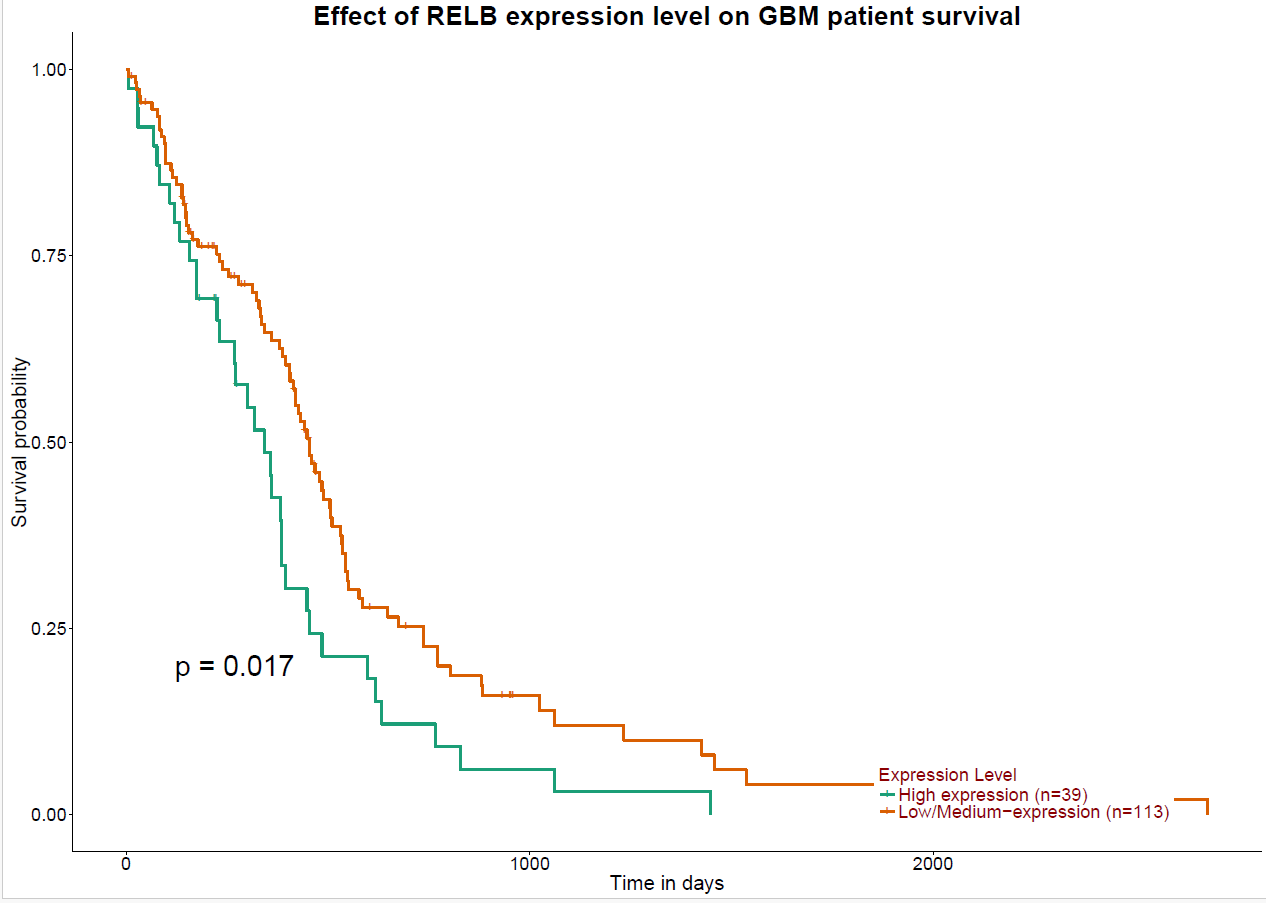


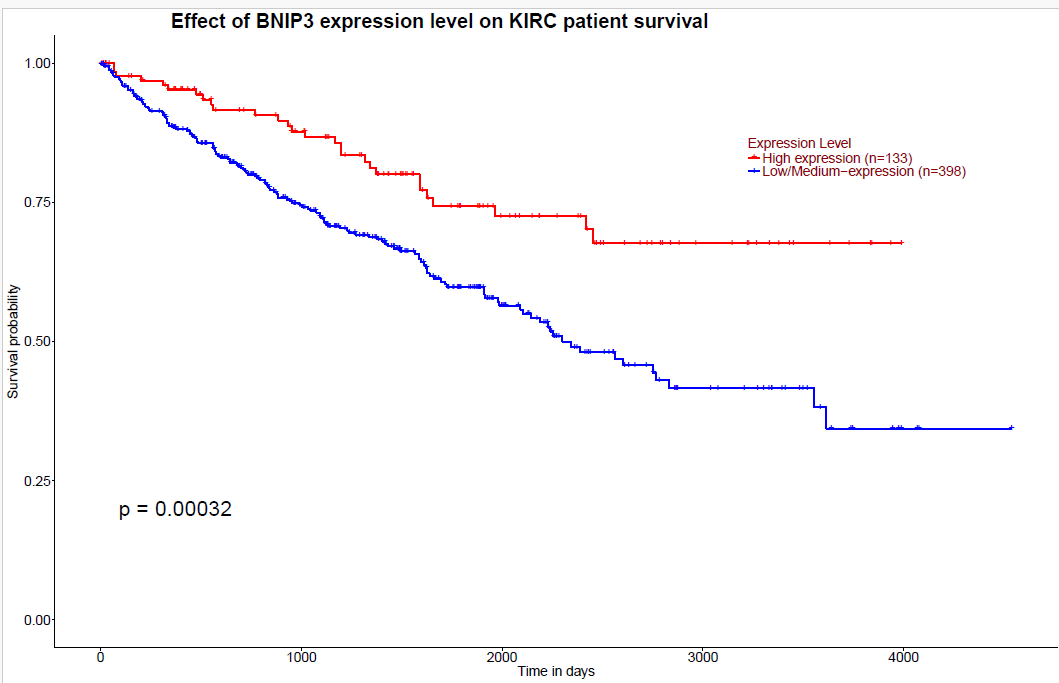

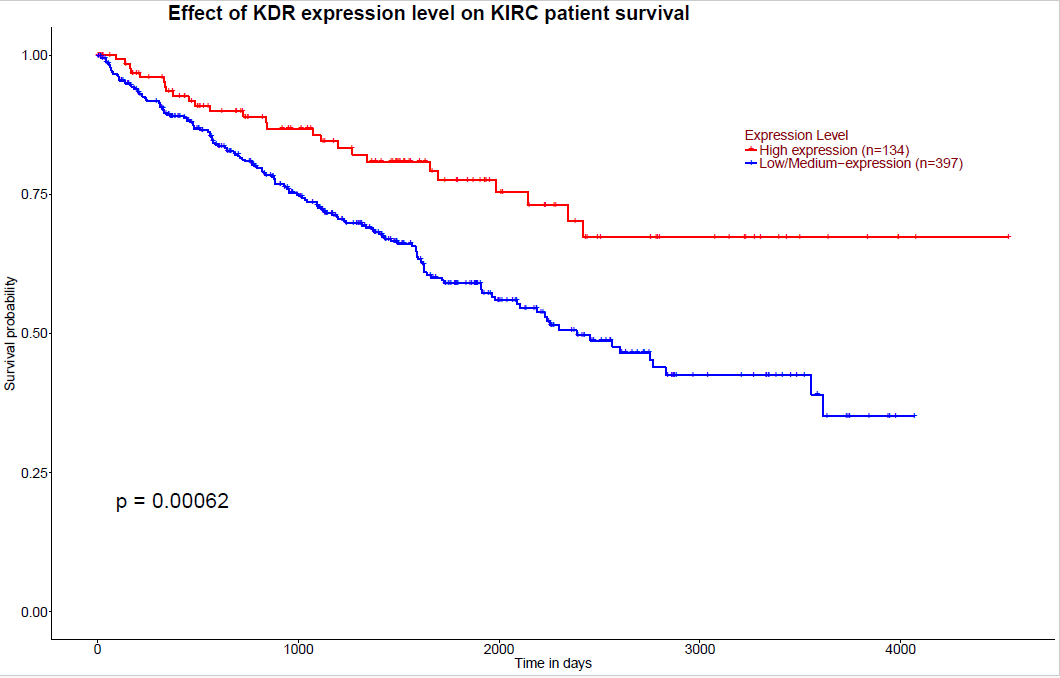

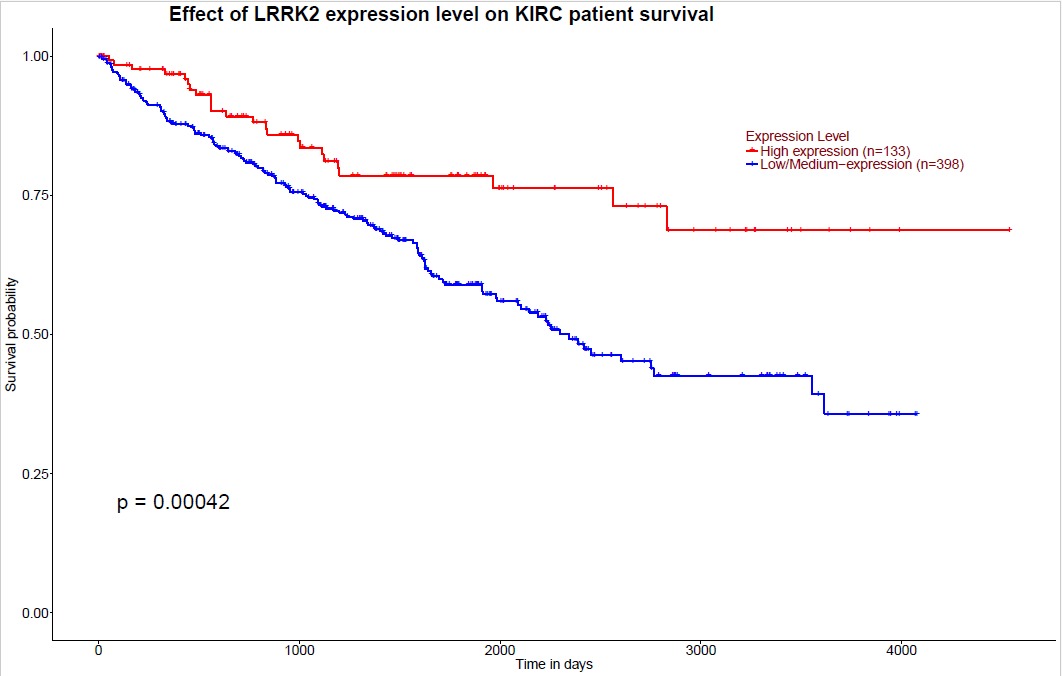


**Supplementary Figure 9.** .

## Supplementary Table

| **Cancer** | **Positive correlation** | **Negative correlation** | **High-expression ATGs** | **Low-expression ATGs** | **Good prognosis (p<0.05)** | **Poor prognosis (p<0.05)** |
| --- | --- | --- | --- | --- | --- | --- |
| GC | HIF1A, SEC23A, CXCR4 | NA | LAMP2, MAPK1, HIF1A, SEC23A, CXCR4, TRAF3, TBC1D5, MAP1LC3B, SPHK1, UBQLN2, RAB25 | GABARAPL | NA | CXCR4 SEC23A |
| GBM | EDEM1, P4HB, HSPA5, ABL2, WIPI1, MCL1, ARNT, SEC23A, CTSB, EPAS1, STAT3, ARSB, ATF6, NRBF2, ERN1, GAA, VDAC1, TM9SF1, CASP8, MTDH, HIF1A, XBP1, PPP1R15A, FKBP1A, RAB13, ITPR1, EEF2K, LAMP1, RELB, PTPN2, TRAF3 | NA | FAS, HSPA5, CASP8, FKBP1A, XBP1, HIF1A, TM9SF1, PGK1, RELB, CCL2, DAP, ARSB, CTSD, ATF6, TNFSF10, CXCR4, CDKN1A, DRAM1, CASP1, MCL1, RAB13, MTDH, FBXO32, DIRAS3, CTSB, STAT3, RGS19, P4HB, LAMP1, CASP4, SERPINA1, BNIP1, STX8 | DENND3, ITPR1, MAPT | NA | HSPA5 P4HB RAB13 RELB |
| LGG | STAT3, EDEM1, KDR, ERN1, TM9SF1, DCN, HSPA5, FKBP1A, EIF2AK3, APOL1, PRKAA1, SEC23A | NA | HSPA5, EIF2AK2, BAX, AURKA, MUL1, SH3GLB1, XBP1, HMOX1, TM9SF1, SEC23B, DAP, ARSB, ATF6, RAB32, VAMP8, CXCR4, CANX, KDR, TRIM5, DRAM1, CASP1, RB1, AKT1, RAB13, MTDH, CAPN2, WDR41, CASP3, CTSB, VCP, RAB8A, FADD, STAT3, RGS19, EIF2AK3, P4HB, LAMP1, SERPINA1 | DIRAS3 | NA | STAT3, EIF2AK3, HSPA5, TM9SF1 |
| LUSC | KDR，ARSB，NLRP3，EDEM1，CTSD，RAB8B，NLRC4，SEC23A | NA | NA | NLRC4, EPAS1, STBD1, GLIPR2, KDR, DRAM1, MCL1, ITPR1, NLRP3, CAPN2, FYCO1, RAB8B, PRKCB, ERN1, TMEM150B, LRRK2, SERPINA1 | NA | NA |
| ccRCC | KDR, EPAS1, SH3GLB1, FKBP1A, GNAI3, RAB8B, ABL2, STAT3, ARNT, RB1, BNIP3L, ATG4C, NEDD4, HDAC1, SIRT1, CALCOCO2, SEC23A, CCNY, HMGB1, TRIM5, PRKAA1, GOPC, CDKN1B, TRAF6, UVRAG, PIP4K2A, PTEN, NBR1, ATF6, MAPK1, FOXO3, MAPK3, CLOCK, PIK3CA, PIK3C2A, RPS6KB1, TECPR2, TBC1D5, MAPK8, EIF2AK2, SNX7, MCL1, PRKAR1A, WDFY3, BECN1, EDEM1, RAB33B, SNX18, CANX, CAMKK2, EI24, MTDH, ST13, PTENP1, MTMR3, TP53, FAS, ATG2B, STX17, RAB12, ULK2, SMCR8, USP33, BCL2, MYC, RAB23, STX7, MAPK9, PGK1, EEF2K, TM9SF1, CASP8, BNIP3, ARSB, USP10, AKT1, UBQLN2, RAB5A, EIF2S1, PRKAA2, TRIM13, RB1CC1, TBK1, TECPR1, PDPK1, STBD1, DAPK1, OPTN, LRRK2, RRAGA, HSP90AB1, PRKDC, FOXO1, PSEN1, PINK1, PIK3C3, PLEKHM2, TP53INP2, RAB1A, PARP1, UBQLN1, ATP13A2, MFN2, TRAF3, ZKSCAN3, EIF2AK3, SNAP29, AMBRA1, ATG5, ANXA7, VCP, MAPK10, ATG12, WDR41, LAMP1, HIF1A, GSK3B, MTOR, DAB2IP, ATG3 | NA | FAS, PGK1, BNIP3L, KDR, MYC, BNIP3, MAPT, LRRK2 | NA | BNIP3, BNIP3L, KDR, LRRK2 | NA |
| CESC | KDR, FOXO1 | NA | NA | KDR, FOXO1 | NA | NA |

## Supplementary Table 3 ATGs significantly correlated with NRP1 prognosis in cancer samples.
